# Surface layer proteins from virulent *Clostridium difficile* ribotypes exhibit signatures of positive selection with consequences for innate immune response

**DOI:** 10.1186/s12862-017-0937-8

**Published:** 2017-03-23

**Authors:** Mark Lynch, Thomas A. Walsh, Izabela Marszalowska, Andrew E. Webb, Micheál MacAogain, Thomas R. Rogers, Henry Windle, Dermot Kelleher, Mary J. O’Connell, Christine E. Loscher

**Affiliations:** 1 Immunomodulation Research Group, School of Biotechnology, Dublin City University, Glasnevin, Dublin 9, Ireland; 2 Bioinformatics and Molecular Evolution Group, School of Biotechnology, Dublin City University, Glasnevin, Dublin 9, Ireland; 3 Department of Clinical Microbiology, Trinity College Dublin, St James Hospital Dublin, Dublin, Ireland; 4 Institute of Molecular Medicine, Trinity College Dublin, Dublin, Ireland; 5 Faculty of Medicine, Imperial College London, London, SW7 2AZ UK; 6 Computational and Molecular Evolutionary Biology Research Group, School of Biology, Faculty of Biological Sciences, The University of Leeds, Leeds, LS2 9JT UK

**Keywords:** *Clostridium difficile*, Surface layer protein, Positive selection, Protein functional shift, Protein evolution, Virulence

## Abstract

**Background:**

*Clostridium difficile* is a nosocomial pathogen prevalent in hospitals worldwide and increasingly common in the community. Sequence differences have been shown to be present in the Surface Layer Proteins (SLPs) from different *C. difficile* ribotypes (RT) however whether these differences influence severity of infection is still not clear.

**Results:**

We used a molecular evolutionary approach to analyse SLPs from twenty-six *C. difficile* RTs representing different *slpA* sequences. We demonstrate that SLPs from RT 027 and 078 exhibit evidence of positive selection (PS). We compared the effect of these SLPs to those purified from RT 001 and 014, which did not exhibit PS, and demonstrate that the presence of sites under positive selection correlates with ability to activate macrophages. SLPs from RTs 027 and 078 induced a more potent response in macrophages, with increased levels of IL-6, IL-12p40, IL-10, MIP-1α, MIP-2 production relative to RT 001 and 014. Furthermore, RTs 027 and 078 induced higher expression of CD40, CD80 and MHC II on macrophages with decreased ability to phagocytose relative to LPS.

**Conclusions:**

These results tightly link sequence differences in *C. difficile* SLPs to disease susceptibility and severity, and suggest that positively selected sites in the SLPs may play a role in driving the emergence of hyper-virulent strains.

**Electronic supplementary material:**

The online version of this article (doi:10.1186/s12862-017-0937-8) contains supplementary material, which is available to authorized users.

## Background

*Clostridium difficile* is a spore-forming, anaerobic gram-positive bacterium and the leading cause of antibiotic-associated diarrhoea worldwide [1]. Infection usually occurs in hospitalised patients receiving broad-spectrum antibiotics [2, 3]. Like many bacteria, *C. difficile* possesses an S-Layer [4, 5] which is proposed to have functions such as adherence and evasion of the immune system [6]. The S-Layer of *C. difficile* is composed of two surface layer proteins (SLPs), termed high molecular weight (HMW) SLP and low molecular weight (LMW) SLP, and is encoded for by a single gene, *slpA*, forming an slpA protein precursor [7–9].

The HMW SLP is highly conserved in *C. difficile*, with up to 97% sequence similarity between strains [7]. The protein exhibits strong and specific binding to gastrointestinal tissues and human epithelial cells [10]. It has been shown that the HMW protein is most likely anchored to the cell wall, and “displays” the LMW protein to the external environment [11]. The LMW SLP exhibits greater sequence variation between strains and, as the outermost component of the organism, is likely region exposed most to the host immune system. This high level of sequence variability observed in the LMW region of the S-layer is not surprising given the evolutionary forces exerted by host defences in response to infection [12]; however evidence that such sequence differences in the LMW region influence the interaction of SLPs with the host is lacking.

Recently, there have been conflicting reports regarding the predictability of severity of infection based on *C. difficile* ribotype (RT) [13–15]. The prevalence of severe and recurrent disease in response to “hyper-virulent” RTs such as 027 and 078 [16–18], while other common RTs such as 001 are not associated with increased virulence, suggests a potential link between ribotype and infection severity. These strains exhibit increased antibiotic and disinfectant resistance [19, 20] increased sporulation rates [21] and other possible modes of action for virulence [22]. Another recent study has shown antibody raised against slpA from *C difficile* strain 630 (PCR ribotype 012) does not cross react with slpA from ribotype 027 [23]. Despite slpA being examined as a vaccine candidate [24], problems may still arrive due to high sequence variability of the protein coding sequences for SLPs between strains. We propose that SLPs from different strains of *C. difficile* may be undergoing different selective regimes, and that this variability can induce variable immune responses with consequences for the observed spectrum of severity of clinical symptoms.

Previously, we demonstrated a role for TLR4 in the host response to *C. difficile* [25]. Specifically, we showed that SLPs from RT 001 activated TLR4 signalling, inducing the maturation of dendritic cells in vitro and subsequent T helper cell activation [25]. More recently, we have shown that 001 SLPs induce clearance responses in macrophages [26] and other studies have also shown SLPs from RT 001 to effectively induce an immune response [27, 28]. Together these findings provide a mechanism for interaction between host and pathogen. However, the influence of SLP sequence on the host immune response is currently unknown, and it is possible that sequence variation may modulate inflammatory response. Here we pose the question: Does variation in SLP sequence play a role in the severity of *C. difficile* infection?

In this study we determine if the vast spectrum of symptoms from mild to severe that are observed across different RTs of *C. difficile* could result from modulation of the immune response caused by sequence variation in the *slpA* gene that codes for the SLPs. We also explored the possibility that SLPs from specific ribotypes are under positive selection (synonymous with protein functional shift), all of which may affect the overall disease severity.

## Results

### The relationship between SLPs of different ribotypes can be depicted on a robust Phylogenetic Tree

**Fig. 1:**
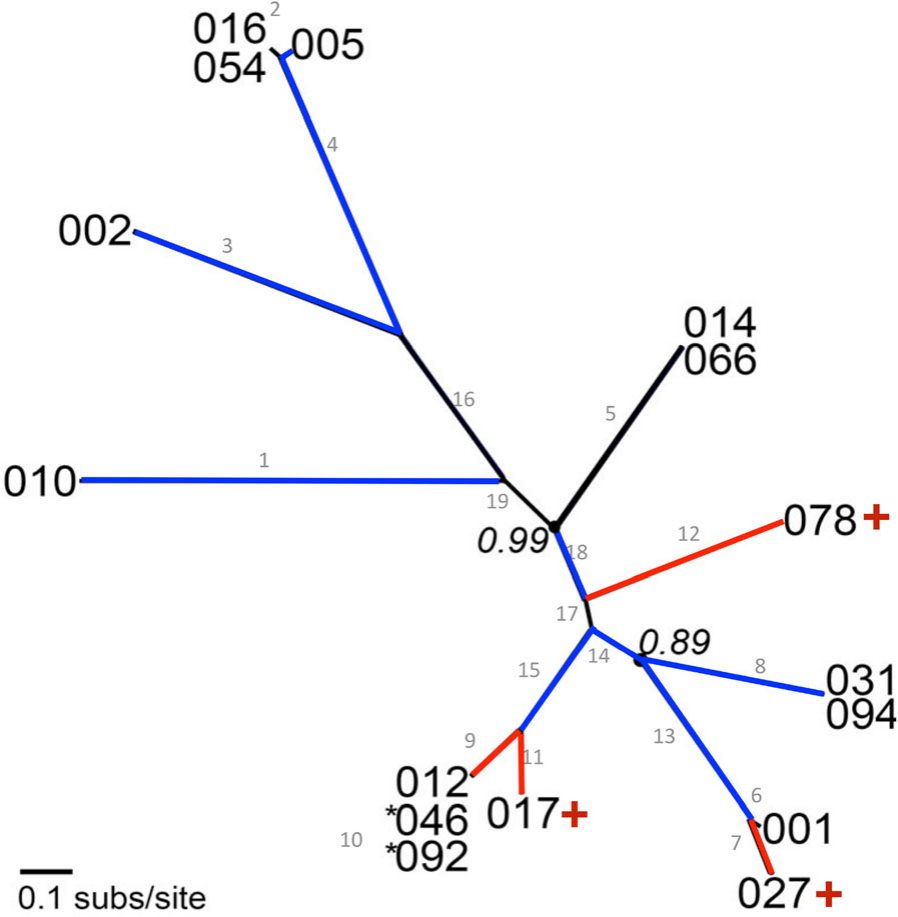
Phylogenetic tree of the slpA protein from 26 strains/16 major ribotypes of *C. difficile.* Each leaf on the tree refers to a specific ribotype, these are named by ribotype number as per convention. The branch numbering scheme is shown in grey. Ribotypes with very short branch lengths are given as clusters at the tips of branches. All but three nodes had posterior probability value (PP) = 1.00: two of these values are shown in italics on the tree (0.99 and 0.89). The final exception was the node joining ribotypes 046 and 092, where * denotes PP of 0.82. Lineages under positive selection are highlighted as follows; blue corresponds to positively selected sites detected in the HMW protein, and red corresponds to positively selected sites in the LMW protein. Where known, the virulence/severity of disease associated with each ribotype is denoted by a red “+” for hypervirulence

Fully annotated *slpA* sequences were taken from previously published studies [8, 29]. MUSCLE [30] was used to generate a phylogenetic tree of all sequences in our dataset (Additional file 1). Likelihood mapping tests were carried out on our alignment of 26 RT *slpA* genes. The results confirmed that sufficient phylogenetic signal existed in the dataset to generate a gene tree for *slpA* (Additional file 1). The *slpA* gene tree was reconstructed using MrBayes v 3.2.1 [31] and visualised using Dendroscope [32] (Fig. 1). The phylogeny shows that the *slpA* sequence from hyper-virulent RT 027 ribotype is closely related to that of RT 001.

### SLPs from different ribotypes of *C. difficile* have evolved under different selective regimes, with highly virulent strains exhibiting signatures of protein functional shift

**Fig. 2:**
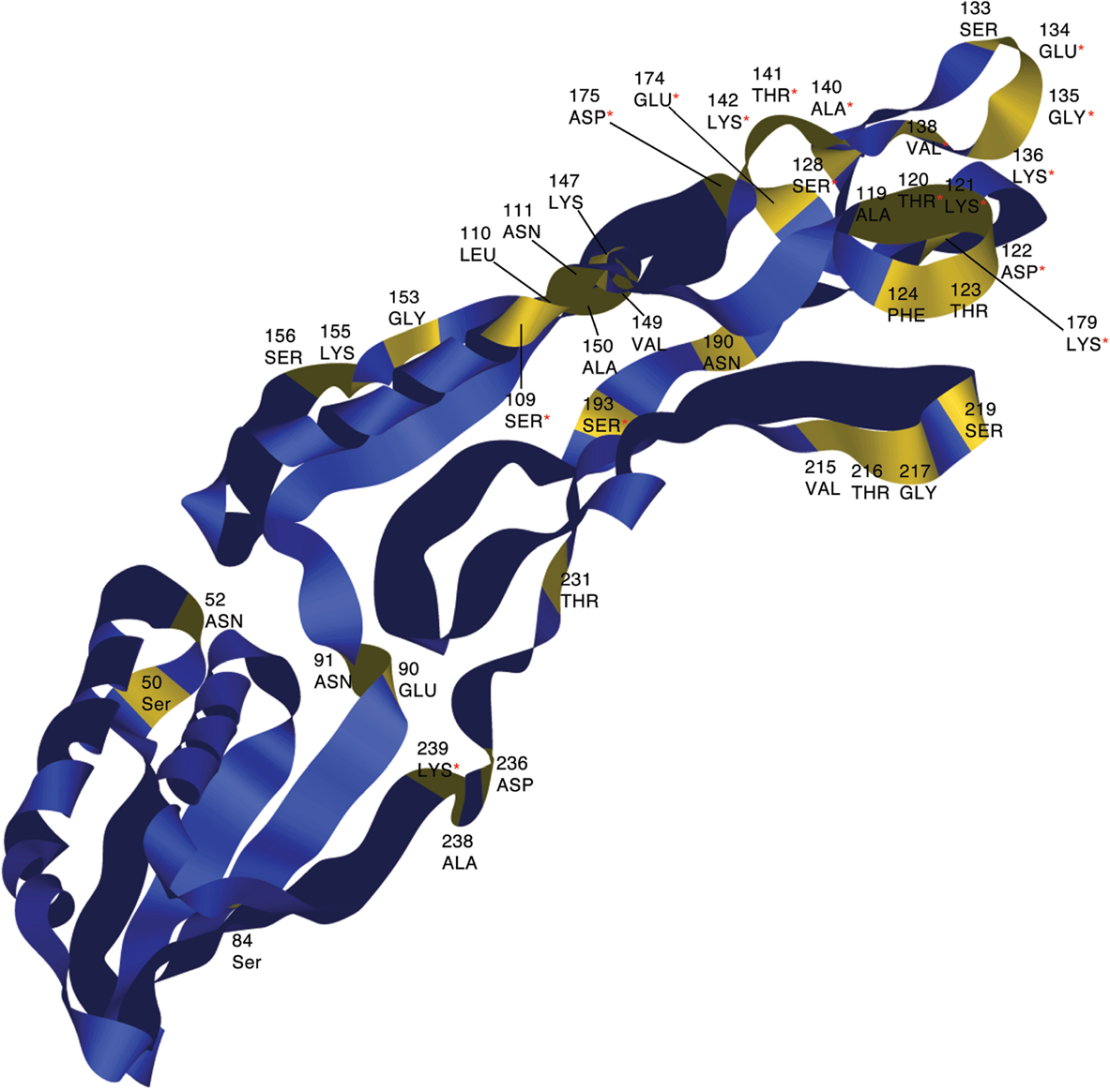
Structural model of slpA LMW sub-unit with positively selected sites highlighted. 3D model of the LMW SLP obtained from PDB (3cvz). α-helices and β-sheets can clearly be seen along with loop regions. Specific amino acids under positive selection are labelled in gold. The majority of sites under positive selection can be seen in Domain 2, which is rich in loops. A red asterisk indicates a residue with a probability of greater than 0.9

We performed two types of analysis on the *slpA* gene alignment and phylogeny to determine heterogeneous selective pressures: firstly we examined variation in selective pressures at the level of sites across the alignment (Table 1), and secondly at the level of lineage/ribotype and site combined (results shown in Table 2 and summarised on Fig. 2) [33]. All Likelihood Ratio Tests performed were standard for these models. The portion of the alignment representing the LMW protein-coding region was highly variable between strains. Under the most statistically significant model, 44 amino acid sites were estimated to have undergone positive selection. As visualised on the 3-D model of LMW SLP these sites are largely located within a loop-rich region in domain 2 (Fig. 2).

**Table 1:** Results of site-specific positive selection analysis of the *slpA* gene

Model	P	lnL	Estimates of parameters	PS sites
M0: one ratio	1	−16877.369043	ω = 0.1440	None
M1: Neutral	1	−16292.610806	p_0_ = 0.61757, p_1_ = 0.38243 ω_0_ = 0.08215, ω_1_ = 1.00000	Not allowed
M2: Selection	4	−16242.808880	p_0_ = 0.59388, p_1_ = 0.32797 (p_2_ = 0.07815), ω_0_ = 0.08640, ω_1_ = 1.0000, ω_2_ = 68.75381	BEB 46 > 0.50 12 > 0.95 4 > 0.99
M3: Discrete (K = 2)	3	−16210.1011	p_0_ = 0.46054, (p_1_ = 0.53946) ω_0_ = 0.03195, ω_1_ = 0.35512	None
M3: Discrete (K = 3)	5	−16098.846498	p_0_ = 0.38293, p_1_ = 0.51573, p_2_ = 0.10134 ω_0_ = 0.01892, ω_1_ = 0.24549, ω_2_ = 20.89735	None
M7: Beta	2	−16105.477356	p = 0.41836, q = 1.29859	Not allowed
M8: Beta&Omega > 1	4	−16052.155863	p_0_ = 0.92067, p = 0.47615, q = 1.96064 (p_1_ = 0.07933), ω = 29.20962	BEB 44 > 0.50 11 > 0.95 4 > 0.99
M8a: Beta&Omega = 1	3	−16095.794065	p_0_ = 0.87118, p = 0.52389, q = 2.94963 (p_1_ = 0.12882), ω = 1.0000	Not allowed

The table shows the number of residues predicted to be under positive selection for each site-specific model in the analyses. All models tested are displayed, along with number of parameters (**P**), log likelihood scores (**lnL**), estimates of parameters (where p = proportion of sites under a particular omega value, and ω = the ratio of non-synonymous substitution per non-synonymous site to synonymous substitution per synonymous site (D_N_/D_S_)). The number of positively selected sites with a given posterior probability is also shown

**Table 2:** Results of lineage-specific positive selection analysis of the *slpA* gene

Branch	Model	P	lnL	Estimation of parameters	PS Sites
1	A	3	−16283.290	p_0_ = 0.612 p_1_ = 0.379 p_2_ = 0.004 p_3_ = 0.002 Background: ω_0_ = 0.081 ω_1_ = 1.000 ω_2_ = 1.000 Foreground: ω_0_ = 0.081 ω_1_ = 1.000 ω_2_ = 999.0	BEB 3 > 0.99
2	A	3	−16287.406	p_0_ = 0.613 p_1_ = 0.379 p_2_ = 0.004 p_3_ = 0.002 Background: ω_0_ = 0.081 ω_1_ = 1.000 ω_2_ = 1.000 Foreground: ω_0_ = 0.081 ω_1_ = 1.000 ω_2_ = 999.0	BEB 3 > 0.99
3	A	3	−16273.156	p_0_ = 0.546 p_1_ = 0.325 p_2_ = 0.080 p_3_ = 0.047 Background: ω_0_ = 0.077 ω_1_ = 1.000 ω_2_ = 1.000 Foreground: ω_0_ = 0.078 ω_1_ = 1.000 ω_2_ = 305.086	BEB 52 > 0.50 11 > 0.95 1 > 0.99
4	A	3	−16254.536	p_0_ = 0.534 p_1_ = 0.326 p_2_ = 0.086 p_3_ = 0.052 Background: ω_0_ = 0.076 ω_1_ = 1.000 ω_2_ = 1.000 Foreground: ω_0_ = 0.078 ω_1_ = 1.000 ω_2_ = 49.513	BEB 51 > 0.50 21 > 0.95 8 > 0.99
7	A	3	−16267.493	p_0_ = 0.577 p_1_ = 0.364 p_2_ = 0.035 p_3_ = 0.022 Background: ω_0_ = 0.082 ω_1_ = 1.000 ω_2_ = 1.000 Foreground: ω_0_ = 0.082 ω_1_ = 1.000 ω_2_ = 999.000	BEB 15 > 0.50
8	A	3	−16286.517	p_0_ = 0.599 p_1_ = 0.369 p_2_ = 0.019 p_3_ = 0.012 Background: ω_0_ = 0.080 ω_1_ = 1.000 ω_2_ = 1.000 Foreground: ω_0_ = 0.080 ω_1_ = 1.000 ω_2_ = 65.618	BEB 9 > 0.50 1 > 0.95
9	A	3	−16280.189	p_0_ = 0.585 p_1_ = 0.366 p_2_ = 0.029 p_3_ = 0.018 Background: ω_0_ = 0.080 ω_1_ = 1.000 ω_2_ = 1.000 Foreground: ω_0_ = 0.081 ω_1_ = 1.000 ω_2_ = 998.998	BEB 11 > 0.50 1 > 0.95
11	A	3	−16289.170	p_0_ = 0.593 p_1_ = 0.372 p_2_ = 0.020 p_3_ = 0.013 Background: ω_0_ = 0.082 ω_1_ = 1.000 ω_2_ = 1.000 Foreground: ω_0_ = 0.081 ω_1_ = 1.000 ω_2_ = 998.975	BEB 3 > 0.50
12	A	3	−16288.187	p_0_ = 0.604 p_1_ = 0.367 p_2_ = 0.017 p_3_ = 0.010 Background: ω_0_ = 0.080 ω_1_ = 1.000 ω_2_ = 1.000 Foreground: ω_0_ = 0.080 ω_1_ = 1.000 ω_2_ = 998.952	BEB 6 > 0.50 1 > 0.95
13	A	3	−16280.744	p_0_ = 0.601 p_1_ = 0.341 p_2_ = 0.036 p_3_ = 0.020 Background: ω_0_ = 0.079 ω_1_ = 1.000 ω_2_ = 1.000 Foreground: ω_0_ = 0.079 ω_1_ = 1.000 ω_2_ = 32.021	BEB 18 > 0.50 1 > 0.99
14	A	3	−16287.185	p_0_ = 0.606 p_1_ = 0.376 p_2_ = 0.010 p_3_ = 0.006 Background: ω_0_ = 0.079 ω_1_ = 1.000 ω_2_ = 1.000 Foreground: ω_0_ = 0.081 ω_1_ = 1.000 ω_2_ = 67.858	BEB 3 > 0.50 2 > 0.95
15	A	3	−16278.296	p_0_ = 0.598 p_1_ = 0.354 p_2_ = 0.029 p_3_ = 0.017 Background: ω_0_ = 0.077 ω_1_ = 1.000 ω_2_ = 1.000 Foreground: ω_0_ = 0.078 ω_1_ = 1.000 ω_2_ = 8.993	BEB 12 > 0.50 4 > 0.95 1 > 0.99
18	A	3	−16277.205	p_0_ = 0.560 p_1_ = 0.337 p_2_ = 0.063 p_3_ = 0.038 Background: ω_0_ = 0.078 ω_1_ = 1.000 ω_2_ = 1.000 Foreground: ω_0_ = 0.077 ω_1_ = 1.000 ω_2_ = 94.127	BEB 45 > 0.50 7 > 0.95 2 > 0.99

The table shown the number of residues predicted to be under positive selection for each site-specific model in the analyses. All models tested are displayed, along with number of parameters (**P**), log likelihood scores (**L**), estimates of parameters (where p = proportion of sites under a particular omega value, and ω = the ratio of non-synonymous substitution per non-synonymous site to synonymous substitution per synonymous site (D_N_/D_S_). The number of positively selected sites with a given posterior probability is also given

The lineage site-specific analyses yielded a more complex story (Table 2). Positive selection was detected in the HMW protein-coding region on a number of specific lineages, an area of the gene that is highly conserved across the strains in the dataset (Additional file 1: Figure S1). In total, eight branches show signatures of positive selection in the HMW protein, including RTs 010, 002, 005, 031 and 094. These RTs have been under selective pressure to adapt, and given the function of the HMW protein, we speculate that the selective pressure at play here may have been for improved adhesion to the host epithelium. Also there were 4 lineages (lineage numbers 7, 9, 11 and 12) that showed evidence of positive selection in the LMW protein. There are relatively few sites in the LMW region under positive selection for these lineages. Of particular interest here are the results for the LMW region on branch 7, leading to RT 027 (Table 2). RT 027 is of clinical importance due to the fact that it is hyper-virulent, and the presence of positively selected residues in the LMW region of its SLP may be a contributing factor to its increased pathogenesis. There was no evidence of positive selection in either LMW or HMW regions in the most common RT 001 or indeed in RT 014.

Two potential recombination events were detected in the *slpA* sequence alignment (Table 3). The first was between RT 017 and 012, with a P-value of 4.98 x 10^−6^. This event corresponds to position 1–33 in the MSA, i.e., almost completely within the signal peptide, and does not overlap with our signatures for positive selection. The second signal for recombination was detected between RT 001 and 027 between positions 174 and 209 on the MSA, with a P-value of 3.44 x 10^−6^. This region of the alignment does indeed encompass several positively selected sites. Caution must therefore be taken in interpreting these particular sites, however many other positively selected sites have been detected outside of this region.

**Table 3:** Results of recombination analysis on the slpA MSA

Ribotype	Parent Sequence	Recombination Detection Method	*P*-Value	Amino Acid position
RT 017	RT 012	RDB	4.98 x 10^−6^	1 – 33
RT 027	RT 001	RDB	3.44 x 10^−6^	174 – 209

Recombination events detected using RDP v3.44 (Martin and Rybicki, 2000) are displayed. Details of ribotype; the ribotype of the parent sequence with which recombination has occurred; detection method; P-value, and the position of the recombination event in the multiple sequence alignment are all given

### SLPs from different ribotypes of *C. difficile* have differential effects on the production of cytokines by macrophages

**Fig. 3:**
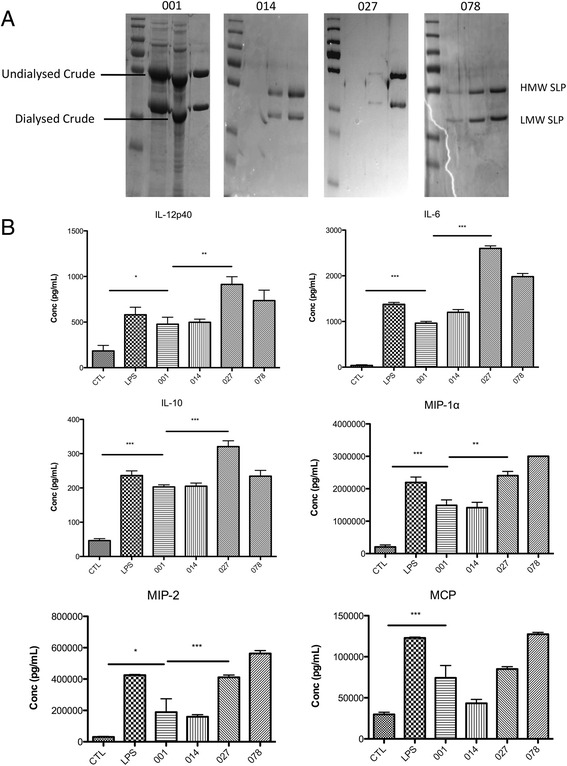
SLPs purified by FPLC induce variable cytokine secretion in macrophages. **a** SLPs from 001, 014, 027 and 078 were purified by FPLC and visualised by SDS-PAGE. Gel images show crude undialysed S-Layer, crude Dialysed S-Layer and purified SLP from ribotype 001. Purified 014, 027 and 078 SLPs can also be seen by the presence of two bands around 32 kDa and 44 kDa, representing the LMW and HMW SLPs respectively. **b** J774 macrophages were stimulated with LPS (100 ng/mL) and SLP (20 μg/mL) from ribotypes 001, 014, 027 and 078 for a period of 24 h, and the control is PBS. Supernatants were collected and cytokine levels were analysed by ELISA. The two horizontal lines represent statistical significance between control cells and 001-stimulated cells, and between 001-stimulated and 027-stimulated cells respectively. The results show the mean (±SEM) for *n* = 3. *** *p* < 0.001, ** *p* < 0.01 and * *p* < 0.05 determined by one-way ANOVA test comparing all groups, with a Newman-Keuls post test, comparing all pairs of columns

We tested our hypothesis that RT-specific sequence differences in SLP influences the immune response by choosing the following 4 samples: RTs 027 and 078 that have a number of sites under positive selection, and RTs 001 and 014 could not find any positive selection acting on the *slpA* gene. We purified the SLPs of these 4 ribotypes from clinical isolates of *C. difficile* (Fig. 3a), sequenced them to confirm they were identical to the samples from the database, and investigated their effects on the production of cytokines by macrophages. Sterile PBS was added to the macrophages as a negative control. Exposure of macrophages to SLPs from RT 001 resulted in the production of levels of IL-12p40, IL-10 and IL-6 that were similar to Lipopolysaccharide (LPS) (Fig. 3b). The cytokine levels induced by RT 014 were almost identical to RT 001. Interestingly, activation of macrophages with RTs 027 and 078 SLPs consistently induced higher levels of IL-12p40, IL-10 and IL-6 and in the case of IL-12p40, a two-fold increase was observed relative to RT 001 (Fig. 3b; * *p* < 0.05; ** *p* < 0.01, *** *p* < 0.001). Furthermore, RTs 027 and 078 also induced higher levels of the chemokines MIP-1α, MIP2 and MCP than RTs 001 and 014. Although it is important to note that in the case of RTs 027 and 078 the expression was to a lesser extent than LPS.

### SLPs from different ribotypes of *C. difficile* have differential effects on expression of cell surface markers on macrophages

**Fig. 4:**
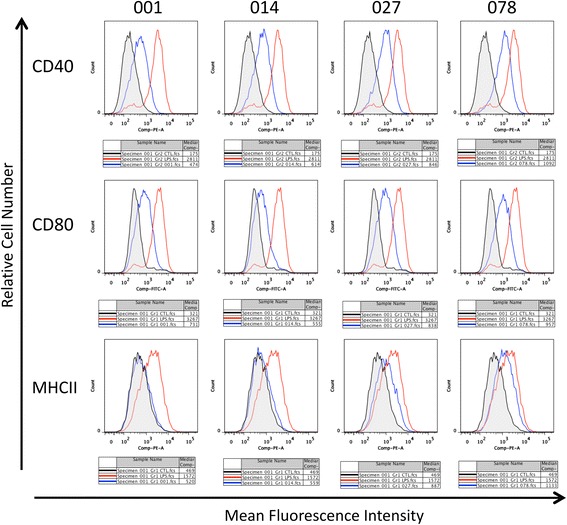
SLPs differentially modulate cell surface marker expression on macrophages. J774 macrophages were stimulated with SLP from ribotypes 001, 014, 027 and 078 (20 μg/mL) for 24 h. LPS (100 ng/mL) was used as a positive control. Results show expression of the cell surface markers CD80 and CD86. Control cells are shaded in grey, SLP-stimulated cells are labelled in blue, and LPS-stimulated cells are labelled in red

Next, we examined the effects of the SLPs on the expression of cell surface markers that are important for antigen presentation and interaction with other immune cells. There was a strong up-regulation of CD40, CD80 and MHC II expression on macrophages in response to LPS (Fig. 4). The SLPs from RTs 001 and 014 also increased expression of CD40, CD80 and MHC II on macrophages, but to a lesser extent than LPS, with RT 014 evoking the weakest response. Cells stimulated with RTs 027 and 078 induced a higher expression of CD40, CD80 and MHC II than either RTs 001 or 014. This increased expression in response to RT 027 and 078 SLP remained less potent than LPS stimulation.

### SLPs from different ribotypes of *C. difficile* have differential effects on phagocytosis by macrophages

**Fig. 5:**
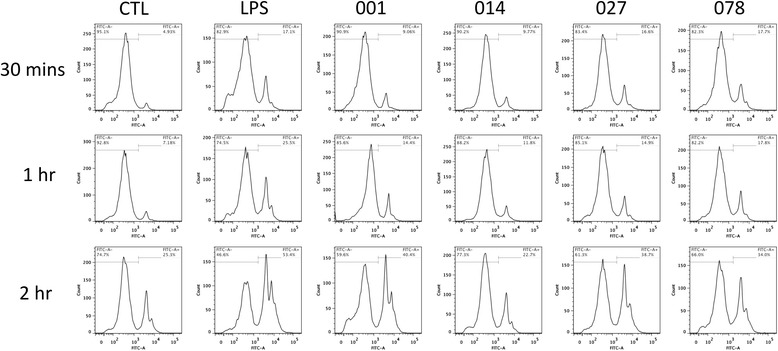
SLPs induce phagocytosis at variable levels in macrophages. Phagocytosis of FITC-labelled fluorescent beads by J774 macrophages in the presence of LPS and SLP ribotypes 001, 014, 027 and 078. Cells were stimulated for 24 h with SLP (20 μg/mL) or LPS (100 ng/mL) and beads were then added (10 beads/cell). Percentage of the cell population was measured by the quantity of FITC signal from the cells using flow cytometry

A key factor in the outcome of infection caused *C. difficile* is the effective clearance of the bacteria; therefore, we next examined the ability of SLPs from the four ribotypes to induce phagocytosis in macrophages. Control cells stimulated with sterile PBS had a low level of phagocytosis at 30 min; less than 5% of the population contained beads (Fig. 5 and Table 4). After 1 h, this had increased marginally to 7% and by 2 h, 25% of cells had phagocytosed the beads. Phagocytosis was significantly increased for LPS-stimulated cells, with 17%, 25% and 53% of macrophages phagocytosing beads at the 30 min, 1 h and 2 h time points respectively. Despite being less potent than LPS, RT 001 SLP also induced phagocytosis. 9%, 14.4% and 40.4% of macrophages were phagocytosing beads at 30mins, 1 h and 2 h respectively. RT 014 SLP induced a similar response to RT 001 SLP at 30mins, with 9.77% of macrophage phagocytosing beads. After 1 h this number had increased to marginally to 11.8%, and 22% of cells were undergoing phagocytosis at 2 h. In contrast, RT 027 and RT 078 SLP-treated cells displayed a similar level of phagocytosis to the LPS controls at 30mins, 16.6% and 17.7% of cells respectively. At 1 h, RT 027 SLP induced phagocytosis at a similar rate to RT 001 (14.9% vs 14.4% respectively, but lower than LPS (25.5%). RT 078 SLP was more potent at this time point, with 17.8% of cells undergoing phagocytosis. At 2 h, RT 027 and 078 SLP were both less potent than RT 001, with RT 027 being marginally lower at 38.7% and RT 078 at 34.0%. Table 4 gives all percentages of phagocytosing cells in response to SLP or LPS.

**Table 4:** The rate of phagocytosis in response to SLP or LPS stimulation

	CTL	LPS	001	014	027	078
30 MIN	4.93	17.1	9.06	9.77	16.6	17.7
1 HR	7.18	25.5	14.4	11.8	14.9	17.8
2 HRS	25.3	53.4	40.4	22.7	38.7	34.0

Table displaying the rate of phagocytosis in response to SLP or LPS stimulation. All numbers are percentages of total macrophage population undergoing phagocytosis

## Discussion and Conclusion

The surface layer proteins of *C. difficile* coat the exterior of the bacterial cell, and are likely the first point of contact with the host immune system. The 26 sequences in our dataset exhibit sequence variation for the *slpA* gene, particularly in the area encoding the LMW protein. In this study we tested for evidence of variation in selective pressure on the SLPs specific to particular RTs. As positive selection has been shown to be synonymous with protein functional shift, we wished to test if this sequence variation, some of which is a result of positive selection on the SLPs, could potentially influence the host response [34, 35].

Our phylogenetic analysis provided us with a sampling strategy for in vitro testing. We detected positive selection on multiple lineages of the *slpA* gene tree, and on both SLP subunits (HMW and LMW). We found sequence signatures of positive selection in the HMW SLP for RTs 002, 005, 010, 031 and 094. This well-conserved region of the gene is involved in binding to the gastrointestinal tract [9] and this result potentially suggests an increased selective pressure for adherence properties in these RTs. Of particular interest were the sites of positive selection detected on the LMW SLP. As previous studies have shown a role for the LMW region in initiating an immune response [25–28], these differences between RTs may affect host recognition of the pathogen. Additionally, we found two hyper-virulent strains, RTs 027 and 078, with positive selection mainly isolated to the LMW subunit.

From our phylogenetic analysis we can see the SLP from RT 027 is most closely related to RT 001, a common strain with moderate severity of infection [8, 36], however RT 027 displays more severe virulence [16]. This poses an interesting question, are there molecular signatures that we can identify in sequence data that may indicate severity of disease? Indeed, we identified a signature of positive selection unique to the RT 027 branch and the majority of positively selected sites in RT 027 are in the LMW region of the *slpA* gene. We also identified positive selection acting on the LMW region of the *slpA* gene on branches leading to RTs 012, 017 and 078. Of these ribotypes, 078 is the best characterised, and was previously associated with hyper-virulence [18, 19].

The majority of sites detected as positively selected were near the outer tip of the protein, an area easily accessible to immune cell receptors. The potential benefit inferred by these amino acid substitutions for the pathogen may be in modulating the host immune response by varying motifs essential for recognition. Given that RTs 027 and 078 are known to be hyper-virulent strains associated with increased inflammation and persistence of infection [18, 37], this sequence variation in the SLPs from RT 027 may affect the host immune response and impact pathogen clearance.

The downstream functions of these observed mutations cannot be predicted in silico, so we attempted to gain a greater understanding of any sequence variation in a series of in vitro experiments. We focused on RTs 001, 014, 027 and 078. Sequence differences exist between these four ribotypes, with positive selection in the *slpA* gene predicted for RTs 027 and 078. We hypothesised that the comparison between these ribotypes would provide insight into the importance of these mutated residues in the ability of SLPs to interact with, and subsequently activate, the immune response.

The ability of SLPs to induce macrophages to produce cytokines and chemokines is an important indicator of how potently they activate the immune system. We have previously shown that RT 001 SLPs activate macrophages and dendritic cells to produce pro-inflammatory cytokines [25, 26] and the profile of cytokines induced was comparable to that of LPS stimulation. In this study we observed that SLPs from different ribotypes elicited distinct responses in macrophages. Of the four ribotypes selected for the in vitro analysis, RTs 001 and 014 did not display any evidence of positive selection in their SLPs. Despite sequence differences existing between the SLPs of these strains, they induce similar responses from macrophages with similar levels of IL-6, IL-10, IL-12p40, MIP-1α, MIP-2 and MCP.

SLPs from RTs 027 and 078 induced a more potent inflammatory response, exhibiting up to two-fold increases in IL-6, IL-12p40 and IL-10 production relative to the SLPs from RTs 001 and 014. Pro-inflammatory IL-12p40 is known for its importance in bacterial clearance, helping to drive a Th1 response in CD4^+^ T cells. Indeed, IL-12p40 knockout mice have been shown to be unable to clear infection of gram negative bacteria *Francisella tularensis* [38]. Conversely, pro-inflammatory IL-6 has been shown to induce tissue damage during bacterial infection [39]. Therefore, the higher levels of these cytokines induced by hyper-virulent RTs 027 or 078 may contribute to increased inflammation and further tissue damage. Chemokine production was also increased by RTs 027 and 078, indicating the potential for enhanced cell recruitment. The ability to recruit immune cells to the site of infection is important in mounting an efficient response to bacterial pathogens [40], however increased macrophage recruitment can also result in inflammation and disease, which has been shown in *C. difficile* infections caused by the RTs 027 and 078 [18, 41]. SLPs from these ribotypes also induced higher levels of the anti-inflammatory IL-10. Given the role of IL-10 in the differentiation of regulatory T cells in suppressing inflammatory responses [42, 43], increased levels of IL-10 may act to impair clearance mechanisms late in inflammation, allowing the bacteria to persist in the gut. IL-10 has previously been shown to block resistance to pathogens [44] and can directly inhibit phagosome maturation [45]. This correlates with our observation that RT 027 and 078 SLPs do not enhance phagocytosis rates relative to RT 001, despite a heighted cytokine response. This may help to further explain the hyper-virulent state of RTs 027 and 078.

We demonstrate that macrophages stimulated with SLPs from RTs 027 and 078 expressed higher levels of CD80, CD40 and MHC II than those induced by either RT 001 or 014. This again provides evidence that SLPs from RTs 027 and 078 induce a more potent inflammatory response in macrophages. Once again we see little difference between the effect of 001 and 014 on the expression of these markers, even though there are sequence differences. The differences we observe in immune response between RT 001 and RT 027 may be influenced by very specific sites in the *slpA* gene. The ability of macrophage to phagocytose invading pathogens is a crucial determinant in clearance of disease [46]. We observed a similar trend in the cells’ ability to phagocytose in response to SLP. The SLPs from our four RTs activated the cells and induced phagocytosis in a similar fashion to LPS. The rate at which cells phagocytosed however varied between ribotypes. SLPs from RT 001 induced the highest rate of phagocytosis relative to LPS. SLPs from RT 014 induced the weakest phagocytic response, in line with the observed minimal cytokine responses. RT 027 SLP induced similar, if marginally lower, levels of phagocytosis relative to RT 001, despite RT 027 SLP being a much more potent inducer of pro-inflammatory cytokines. As previously stated, the 027-induced increase in IL-10 may account for this, rendering them no more efficient at activating macrophages to engulf and destroy the pathogen. The lack of enhanced phagocytosis in response to these potent RT 027 and 078 SLPs, along with increased cytokine production may suggest high levels of inflammation are beneficial in some way to the bacteria. Indeed it has been shown that phagocytosed *C. difficile* spores can readily survive inside the phagosomes of macrophages [47]. This increased inflammatory state in the gut will increase tissue damage, and expose the pathogen to components of the extracellular matrix to which it can bind [10, 48], thereby allowing the bacteria to gain a greater foothold.

To fully understand the significance of the observed differences in immune response between SLPs from different ribotypes, further analyses, including the use of animal models, must be carried out. Further expansion of the library of SLPs available for study will also allow comparisons between more diverse strains. This study clearly highlights the ability of SLPs to induce variable immune responses, and that SLPs purified from “hyper-virulent” strains seem to induce more potent inflammation. The SLPs from hyper-virulent strains (RT 027 and 078) consistently caused macrophage to produce high levels of pro-inflammatory cytokines and cell surface markers. Levels of phagocytosis for these two ribotypes were lower than LPS-induced phagocytosis and comparable to RT 001-induced phagocytosis. This shows that despite greater induction of pro-inflammatory cytokine production, the SLPs from the hyper-virulent ribotypes studied do not activate macrophages to physically clear the bacteria at a greater rate than RT 001.

We have detected evidence for positive selection in the *slpA* gene of several strains of the pathogen, and while we cannot directly correlate positive selection with increased inflammatory potency, the pattern of selective pressure observed warrants further investigation. Additional experimentation examining the effects of site-directed mutagenesis on the predicted sites may elucidate the true role these mutations have on the host immune response. Regardless of the role of positive selection, it is evident that SLPs isolated from these hyper-virulent strains do indeed modulate the host response, potentially for the benefit of the pathogen. Inhibition of clearance will increase and prolong inflammation, resulting in epithelial tissue damage, allowing the pathogen to invade deeper, binding to extracellular matrix components as previously reported [9] and leading to a colitis-type state in the gut, which is frequently observed in severe *C. difficile* infections [49, 50]. These results suggest the importance of SLPs in disease susceptibility and severity, and that positive selection and protein functional shift in the SLP protein may be playing a role in driving the emergence of hyper-virulent strains.

## Methods

### Phylogeny of the *slpA* gene sequences

In total 26 *slpA* gene sequences were obtained from 16 different ribotypes of *Clostridium difficile*. Sequences were taken from previously published studies, and were fully annotated [8, 29]. Multiple sequence alignments (MSAs) were generated using the software package MUSCLE 3.6 [30] and also using ClustalW [51]. As there was no significant difference between the resultant alignments we used the MUSCLE alignment throughout the analysis (Additional file 1: Figure S1). We performed a test for amino acid composition bias on the alignment in TREEPUZZLE 5.2 [52]. A chi-squared test is performed to compare the amino acid composition of each sequence in the dataset to the frequency distribution assumed in the maximum likelihood model. This distribution assumes homogeneity of composition, i.e., no compositional bias present. If compositional bias is present it can result in erroneous placement of taxa, therefore sequences that failed the test were excluded from further analysis. Likelihood Mapping Tests were performed to assess if the data for *slpA* contained sufficient phylogenetic signal to extract an underlying phylogenetic model of vertical descent. Likelihood mapping involves reducing the phylogenetic tree into all possible quartets (groups of 4 taxa) and assessing the support for each possible quartet [52]. If the data contains phylogenetic signal, then the likelihood of all three possible relationships for the taxa in that quartet will be equally likely (this is represented by quartets populating the three vertices). If sufficient phylogenetic signal is present, the majority of the signal will appear in these vertices and will be equally distributed between the three vertices. If little or no phylogenetic signal is present, the majority of the signal will be toward central region of the triangle, representing an unresolved phylogeny or data unsuitable for phylogenetic modelling. An example of the profiles treated as acceptable and unacceptable for the purpose of this study, along with the full output of the tests, can be seen in Supplementary File 1. Modelgenerator v0.85 [53] was used to compare the fit of 88 different models of evolution with the data and to select the model of best fit. The substitution model selected as the best fit model to the data in Modelgenerator was the WAG + G + F model. The phylogenetic tree for *slpA* was estimated using MrBayes v3.2.1 [31]. Optimisation was achieved using the Nearest Neighbour Interchange (NNI) tree search algorithm and 100 bootstrap replicates implemented under the Akaike Information Criterion (AIC) statistic. Clade support values were given as PPs. A test for recombination was carried out for the in *slpA* gene using the Recombination Detection Program (RDP v3.44) [54].

### Analysis of selective pressure variation

Site-specific and lineage-specific models were applied to the data, allowing for ω values to vary across sites and along different branches, i.e. strain-specific. The models differed in their complexity and have been given the conventional naming scheme [55]. Seven site-specific models and two branch-specific models were used. The site-specific models are described first. The first model M0 assumes that the rate of evolution is constant across all sites and lineages, and calculates a single value for ω across the entire alignment. The next model is known as M1 or “the neutral model” and allows for two classes of sites with ω_0_ = 0 and ω_1_ = 1; under this model purifying selection or neutral evolution are allowed, but positive selection is not permitted. Model M2, the selection model, adds more parameters to M1 and allows for three classes of sites, ω_0_ = 1, ω_1_ = 0 and ω_2_ which is estimated entirely from the data (and free to be >1). All associated proportions of sites fitting into each of these categories are estimated from the data. M1 and M2 can be compared to one another in a Likelihood ratio test (LRT), as M2 is an extension of M1. The next model, M3, an extension of M0, allows for additional ω values to be included, the values of which are estimated entirely from the data. This model can allow two classes of sites to vary (k = 2) or three classes of sites to vary (k = 3). An LRT between M3 (k = 2) and M0 can be used and M3 (k = 3) can be compared by LRT directly with M3 (k = 2) [55].

The remaining models are different from those mentioned previously as they use discrete approximations to continuous distributions in order to model variability in ω at different sites across the alignment. M7 gives variation in ω across a beta distribution. Under this model, ten classes of sites are assumed to exist with ω values constrained between zero and one. M8 is a similar model to M7, but it allows for an additional class of site with its ω value estimated entirely from the data and free to be greater than 1. M8 can be compared with M7 in an LRT. A final model, M8a, is the null model of M8. It restricts the additional site category that is estimated from the data to be ω = 1, and therefore does not allow for positive selection.

The two lineage-specific models applied were Model A and Model A null. Model A allows ω to vary across different lineages as well as across sites. Model A is a lineage-specific extension of M1. Model A null does not allow for positive selection; it can be compared by LRT with Model A.

In all models where selection is permitted, the posterior probability (PP) of any given site in the alignment being under positive selection can be estimated using either Naive Empirical Bayes (NEB) or Bayes Empirical Bayes (BEB). NEB has been reported to be more error prone than BEB. False positives are a particular issue with small datasets where ML estimates may have large sampling errors, and so BEB is the preferred estimator [56].

The LRTs detailed above were carried out for each model, the log likelihood (lnL) values were recorded, with the lnL values closest to zero representing a closer fit to the data. χ2 tests were then used to determine the significance of these models using the degrees of freedom given in Table 1.

A 3-D structure of the LMW SLP was obtained from EMBL-EBI (www.ebi.ac.uk) [57]. The PDB code for this structure is 3cvz. It was used to visualise positively selected residues on the protein (Fig. 2).

### Sequencing of *slpA* gene sequences in *C. difficile* clinical isolates

The strains used in this study included R13537 (ribotype 001) and R12885 (ribotype 014). In these strains the sequence of the *slpA* gene has been previously determined (accession numbers DQ060626 and DQ060638 respectively). To determine the *slpA* gene sequences of our clinical strains belonging to ribotypes 027 and 078, whole-genome sequencing was performed. DNA was extracted from *C. difficile* using the Roche High-pure PCR template preparation kit (Roche diagnostics, West Sussex, UK). Nextera XT library preparation reagents (Illumina, Eindhoven, The Netherlands) were used to generate multiplexed sequencing libraries of *C. difficile* genomic DNA, and resultant libraries were sequenced on an Illumina MiSeq®. Short-read data obtained has been deposited in the European Nucleotide Archive (ENA); project accession number PRJEB6566. Genome assemblies were performed using the Velvet short-read assembler [58] and *slpA* gene sequences were retrieved for each isolate using a nucleotide BLAST search (BLASTN 2.6.1+) [59]. The *slpA* sequence for RT 027 showed 100% identity (e-value 0.0) with previously sequenced RT 027 strains R20291 and CD196. The RT 078 *slpA* sequence showed 100% identity (e-value 0.0) with strain HPA R13540, also RT 078, whose *slpA* sequence is DQ060643, already included in our dataset.

### *C. difficile* growth and S-Layer extraction

*C. difficile* (PCR ribotypes 001, 014, 027, 078) isolated from patients with *C. difficile* infection were used for preparation of SLPs as previously described [25]. Briefly, SLPs were purified from cultures grown anaerobically at 37 °C in BHI/0.05% thioglycolate broth. Cultures were harvested and crude SLP extracts dialysed and applied to an anion exchange column attached to an AKTA FPLC system (MonoQ HR 10/10 column, GE Healthcare). The pure SLPs were eluted with a linear gradient of 0–0.3 mol/L NaCl at a flow rate of 4 mL/min and the process was optimised for each individual ribotype. Peak fractions corresponding to pure SLPs were analysed on 12% SDS-PAGE gels stained with Coomassie blue.

## ELISA

J774A.1 macrophages (ECACC, maintained in (RPMI) 1640 media supplemented with 10% (v/v) heat inactivated foetal bovine serum (FBS) and 2% (v/v) Penicillin-Streptomycin) were stimulated with SLPs (20 μg/ml), a negative control PBS, or positive control LPS (100 ng/ml), for 24 h. Culture supernatants were removed and stored at −80 °C until further analysis. IL-6, IL-12p40, TNFα, IL-10, MIP-1α, MIP-2 and MCP concentrations were analysed by DuoSet ELISA Kits (R&D Systems) according to manufacturer’s instructions.

### Flow cytometry

J774A.1 macrophages were stimulated with SLPs (20 μg/ml) or a positive control, LPS (100 ng/ml), for 24 h, then washed and stained with specific antibodies for CD40 (eBiosciences), CD80, CD86 and MHC Class II (Becton Dickinson). Post 30-min incubation at 4 °C, cells were washed and immunofluorescence analysis was performed on a FACSAria. Data was analysed using FlowJo Software (Treestar, San Carlos, CA).

### Phagocytosis assay

J774A.1 macrophages were stimulated with SLP (20 μg/ml) for 24 h. Subsequently 0.5 x 10^6^ FITC labelled latex fluorescent beads (Sigma Aldrich, L4655) were added. 30 min, 1 h and 2 h post addition of beads, cells were washed in FACS buffer. The uptake of beads (λ_ex_ ~470 nm; λ_em_ ~505 nm), indicating the rate of phagocytosis, was measured by flow cytometry.

## Additional file

Additional file 1:
**a** Multiple sequence alignment (MSA) of the *slpA* gene. The MSA were generated using MUSCLE and ClustalX, and included sequences from 26 strains of Clostridium difficile, representing 16 major ribotypes. Areas of the alignment corresponding to both LMW and HMW subunits are highlighted, as are areas essential for binding and complex formation. Putative positively selected residues are shown with an asterix at their location. **b** Results of likelihood mapping in the SLP dataset. In the uppermost triangle, each dot represents the phylogenetic support for each of the possible quartets generated from the data. The two triangles below summarise these results as percentages. In general – the fewer samples in the centre of the triangle, and the more evenly the samples are distributed across the three vertices – the greater the amount of phylogenetic signal. As determined from the figure, the vast majority of signals (>96%) appear in the vertices of the triangle, and they are evenly dispersed amongst all three vertices, indicating there is sufficient phylogenetic signal within the dataset for the analysis to be carried out and for a gene tree to be generated. (PDF 5188 kb)

## Abbreviations

AIC: Aikike Information Criterion
BEB: Bayes Empirical Bayes
HMW: High Molecular Weight
lnL: Log likelihood
LPS: Lipopolysaccharide
LRT: Likelihood ratio test
LWM: Low Molecular Weight
ML: Maximum Likelihood
NEB: Naïve Empirical Bayes
NNI: Nearest neighbor interchange
PP: Posterior probability
PS: Positive Selection
RT: Ribotype
SLP: Surface layer protein
