# Erratum to: Surface layer proteins from virulent Clostridium difficile ribotypes exhibit signatures of positive selection with consequences for innate immune response

**DOI:** 10.1186/s12862-017-0990-3

**Published:** 2017-06-12

**Authors:** Mark Lynch, Thomas A. Walsh, Izabela Marszalowska, Andrew E. Webb, Micheál Mac Aogáin, Thomas R. Rogers, Henry Windle, Dermot Kelleher, Mary J. O’Connell, Christine E. Loscher

**Affiliations:** 1 Immunomodulation Research Group, School of Biotechnology, Dublin City University, Glasnevin, Dublin 9, Ireland; 2 Bioinformatics and Molecular Evolution Group, School of Biotechnology, Dublin City University, Glasnevin, Dublin 9, Ireland; 3 Department of Clinical Microbiology, Trinity College Dublin, St James Hospital Dublin, Dublin, Ireland; 4 Institute of Molecular Medicine, Trinity College Dublin, Dublin, Ireland; 5 Faculty of Medicine, Imperial College London, London, SW7 2AZ UK; 6 Computational and Molecular Evolutionary Biology Research Group, School of Biology, Faculty of Biological Sciences, The University of Leeds, Leeds, LS2 9JT, UK

## Erratum

“Upon publication of the original article [1], it was noticed that there was an error in the author name. The author’s name should be "Micheál Mac Aogáin" instead of Micheál MacAogain.”
