# Supplementary material for: Surface layer proteins from virulent Clostridium difficile ribotypes exhibit signatures of positive selection with consequences for innate immune response
Source: BMC Evol Biol. 2017 Mar 23;17:90. doi: 10.1186/s12862-017-0937-8 (PMC5364705; doi:10.1186/s12862-017-0937-8)
Supplement: Additional file 1: — a Multiple sequence alignment (MSA) of the slpA gene. The MSA were generated using MUSCLE and ClustalX, and included sequences from 26 strains of Clostridium difficile, representing 16 major ribotypes. Areas of the alignment corresponding to both LMW and HMW subunits are highlighted, as are areas essential for binding and complex formation. Putative positively selected residues are shown with an asterix at their location. b Results of likelihood mapping in the SLP dataset. In the uppermost triangle, each dot represents the phylogenetic support for each of the possible quartets generated from the data. The two triangles below summarise these results as percentages. In general – the fewer samples in the centre of the triangle, and the more evenly the samples are distributed across the three vertices – the greater the amount of phylogenetic signal. As determined from the figure, the vast majority of signals (>96%) appear in the vertices of the triangle, and they are evenly dispersed amongst all three vertices, indicating there is sufficient phylogenetic signal within the dataset for the analysis to be carried out and for a gene tree to be generated. (PDF 5188 kb) [file 12862_2017_937_MOESM1_ESM.pdf]

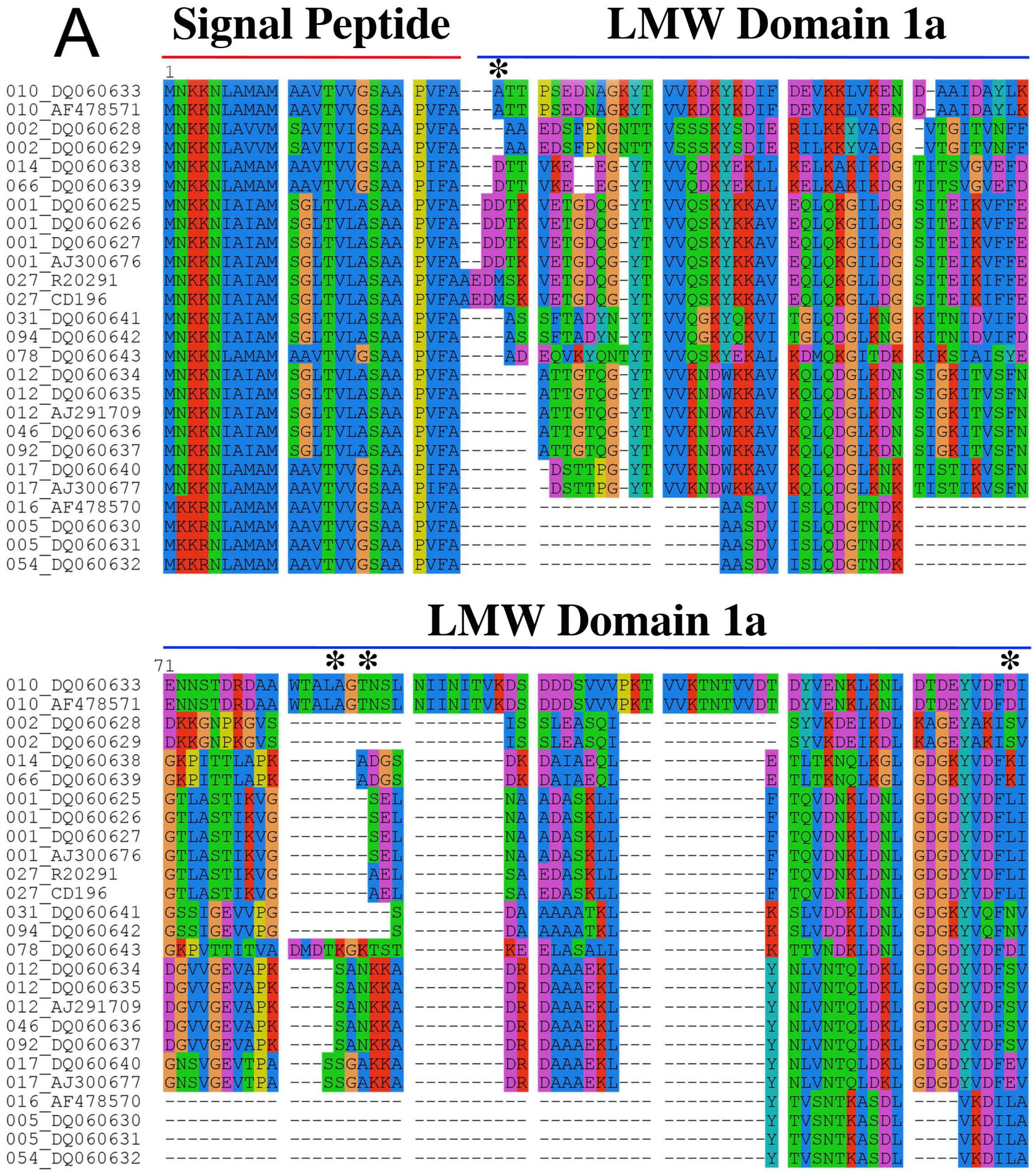
A
Signal Peptide
LMW Domain 1a
1
*
010_DQ060633 MNKKNLAMAM AAVTVVGSAA PVFA---ATT PSEDNAGKYT VVKDKYKDIF DEVKKLVKEN D-AAIDAYLK
010_AF478571 MNKKNLAMAM AAVTVVGSAA PVFA---ATT PSEDNAGKYT VVKDKYKDIF DEVKKLVKEN D-AAIDAYLK
002_DQ060628 MNKKNLAVVM SAVTVIGSAA PVFA----AA EDSFPNGNTT VSSSKYSDIE RILKKYVADG -VTGITVNFF
002_DQ060629 MNKKNLAVVM SAVTVIGSAA PVFA----AA EDSFPNGNTT VSSSKYSDIE RILKKYVADG -VTGITVNFF
014_DQ060638 MNKKNLAMAM AAVTVVGSAA PIFA---DTT VKE--EG-YT VVQDKYEKLL KELKAKIKDG TITSVGVEFD
066_DQ060639 MNKKNLAMAM AAVTVVGSAA PIFA---DTT VKE--EG-YT VVQDKYEKLL KELKAKIKDG TITSVGVEFD
001_DQ060625 MNKKNIAIAM SGLTVLASAA PVFA--DDTK VETGDQG-YT VVQSKYKKAV EQLQKGILDG SITEIKVFFE
001_DQ060626 MNKKNIAIAM SGLTVLASAA PVFA--DDTK VETGDQG-YT VVQSKYKKAV EQLQKGILDG SITEIKVFFE
001_DQ060627 MNKKNIAIAM SGLTVLASAA PVFA--DDTK VETGDQG-YT VVQSKYKKAV EQLQKGILDG SITEIKVFFE
001_AJ300676 MNKKNIAIAM SGLTVLASAA PVFA--DDTK VETGDQG-YT VVQSKYKKAV EQLQKGILDG SITEIKVFFE
027_R20291 MNKKNIAIAM SGLTVLASAA PVFAAEDMSK VETGDQG-YT VVQSKYKKAV EQLQKGLLDG SITEIKIFFE
027_CD196 MNKKNIAIAM SGLTVLASAA PVFAAEDMSK VETGDQG-YT VVQSKYKKAV EQLQKGLLDG SITEIKIFFE
031_DQ060641 MNKKNIAIAM SGLTVLASAA PVFA----AS SFTADYN-YT VVQGKYQKVI TGLQDGLKNG KITNIDVIFD
094_DQ060642 MNKKNIAIAM SGLTVLASAA PVFA----AS SFTADYN-YT VVQGKYQKVI TGLQDGLKNG KITNIDVIFD
078_DQ060643 MNKKNLAMAM AAVTVVGSAA PVFA----AD EQVKYQNTYT VVQSKYEKAL KDMQKGITDK KIKSIAISYE
012_DQ060634 MNKKNIAIAM SGLTVLASAA PVFA------ ATTGTQG-YT VVKNDWKKAV KQLQDGLKDN SIGKITVSFN
012_DQ060635 MNKKNIAIAM SGLTVLASAA PVFA------ ATTGTQG-YT VVKNDWKKAV KQLQDGLKDN SIGKITVSFN
012_AJ291709 MNKKNIAIAM SGLTVLASAA PVFA------ ATTGTQG-YT VVKNDWKKAV KQLQDGLKDN SIGKITVSFN
046_DQ060636 MNKKNIAIAM SGLTVLASAA PVFA------ ATTGTQG-YT VVKNDWKKAV KQLQDGLKDN SIGKITVSFN
092_DQ060637 MNKKNIAIAM SGLTVLASAA PVFA------ ATTGTQG-YT VVKNDWKKAV KQLQDGLKDN SIGKITVSFN
017_DQ060640 MNKKNLAMAM AAVTVVGSAA PIFA------ -DSTTPG-YT VVKNDWKKAV KQLQDGLKNK TISTIKVSFN
017_AJ300677 MNKKNLAMAM AAVTVVGSAA PIFA------ -DSTTPG-YT VVKNDWKKAV KQLQDGLKNK TISTIKVSFN
016_AF478570 MKKRNLAMAM AAVTVVGSAA PVFA------ ---------- -----AASDV ISLQDGTNDK ----------
005_DQ060630 MKKRNLAMAM AAVTVVGSAA PVFA------ ---------- -----AASDV ISLQDGTNDK ----------
005_DQ060631 MKKRNLAMAM AAVTVVGSAA PVFA------ ---------- -----AASDV ISLQDGTNDK ----------
054_DQ060632 MKKRNLAMAM AAVTVVGSAA PVFA------ ---------- -----AASDV ISLQDGTNDK ----------
LMW Domain 1a
71
* * *
010_DQ060633 ENNSTDRDAA WTALAGTNSL NIINITVKDS DDDSVVVPKT VVKTNTVVDT DYVENKLKNL DTDEYVDFDI
010_AF478571 ENNSTDRDAA WTALAGTNSL NIINITVKDS DDDSVVVPKT VVKTNTVVDT DYVENKLKNL DTDEYVDFDI
002_DQ060628 DKKGNPKGVS ---------- --------IS SLEASQI--- ---------- SYVKDEIKDL KAGEYAKISV
002_DQ060629 DKKGNPKGVS ---------- --------IS SLEASQI--- ---------- SYVKDEIKDL KAGEYAKISV
014_DQ060638 GKPITTLAPK ------ADGS --------DK DAIAEQL--- ---------E TLTKNQLKGL GDGKYVDFKI
066_DQ060639 GKPITTLAPK ------ADGS --------DK DAIAEQL--- ---------E TLTKNQLKGL GDGKYVDFKI
001_DQ060625 GTLASTIKVG -------SEL --------NA ADASKLL--- ---------F TQVDNKLDNL GDGDYVDFLI
001_DQ060626 GTLASTIKVG -------SEL --------NA ADASKLL--- ---------F TQVDNKLDNL GDGDYVDFLI
001_DQ060627 GTLASTIKVG -------SEL --------NA ADASKLL--- ---------F TQVDNKLDNL GDGDYVDFLI
001_AJ300676 GTLASTIKVG -------SEL --------NA ADASKLL--- ---------F TQVDNKLDNL GDGDYVDFLI
027_R20291 GTLASTIKVG -------AEL --------SA EDASKLL--- ---------F TQVDNKLDNL GDGDYVDFLI
027_CD196 GTLASTIKVG -------AEL --------SA EDASKLL--- ---------F TQVDNKLDNL GDGDYVDFLI
031_DQ060641 GSSIGEVVPG ---------S --------DA AAAATKL--- ---------K SLVDDKLDNL GDGKYVQFNV
094_DQ060642 GSSIGEVVPG ---------S --------DA AAAATKL--- ---------K SLVDDKLDNL GDGKYVQFNV
078_DQ060643 GKPVTTITVA DMDTKGKTST --------KE ELASALL--- ---------K TTVNDKLDNL GDGDYVDFDI
012_DQ060634 DGVVGEVAPK ----SANKKA --------DR DAAAEKL--- ---------Y NLVNTQLDKL GDGDYVDFSV
012_DQ060635 DGVVGEVAPK ----SANKKA --------DR DAAAEKL--- ---------Y NLVNTQLDKL GDGDYVDFSV
012_AJ291709 DGVVGEVAPK ----SANKKA --------DR DAAAEKL--- ---------Y NLVNTQLDKL GDGDYVDFSV
046_DQ060636 DGVVGEVAPK ----SANKKA --------DR DAAAEKL--- ---------Y NLVNTQLDKL GDGDYVDFSV
092_DQ060637 DGVVGEVAPK ----SANKKA --------DR DAAAEKL--- ---------Y NLVNTQLDKL GDGDYVDFSV
017_DQ060640 GNSVGEVTPA ---SSGAKKA --------DR DAAAEKL--- ---------Y NLVNTQLDKL GDGDYVDFEV
017_AJ300677 GNSVGEVTPA ---SSGAKKA --------DR DAAAEKL--- ---------Y NLVNTQLDKL GDGDYVDFEV
016_AF478570 ---------- ---------- ---------- ---------- ---------Y TVSNTKASDL ----VKDILA
005_DQ060630 ---------- ---------- ---------- ---------- ---------Y TVSNTKASDL ----VKDILA
005_DQ060631 ---------- ---------- ---------- ---------- ---------Y TVSNTKASDL ----VKDILA
054_DQ060632 ---------- ---------- ---------- ---------- ---------Y TVSNTKASDL ----VKDILA

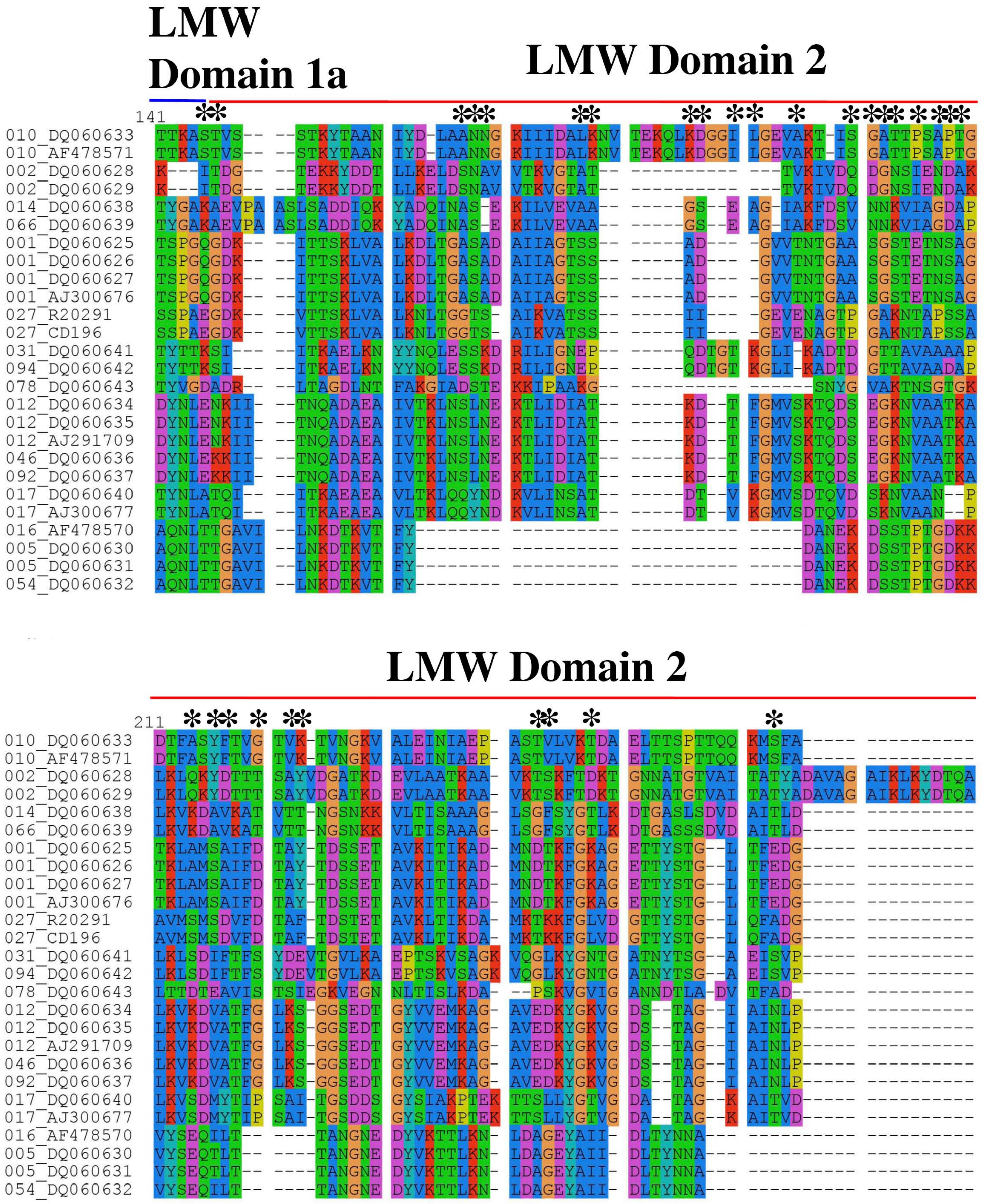
LMW Domain 1a
LMW Domain 2
141
010_DQ060633 TTKASTVS-- --STKYTAAN IYD-LAANNG KIIIDALKNV TEKQLKDGGI LGEVAKT-IS GATTPSAPTG
010_AF478571 TTKASTVS-- --STKYTAAN IYD-LAANNG KIIIDALKNV TEKQLKDGGI LGEVAKT-IS GATTPSAPTG
002_DQ060628 K---ITDG-- --TEKKYDDT LLKELDSNAV VTKVGTAT-- ---------- ---TVKIVDQ DGNSIENDAK
002_DQ060629 K---ITDG-- --TEKKYDDT LLKELDSNAV VTKVGTAT-- ---------- ---TVKIVDQ DGNSIENDAK
014_DQ060638 TYGAKAEVPA ASLSADDIQK YADQINAS-E KILVEVAA-- -----GS--E AG-IAKFDSV NNKVIAGDAP
066_DQ060639 TYGAKAEVPA ASLSADDIQK YADQINAS-E KILVEVAA-- -----GS--E AG-IAKFDSV NNKVIAGDAP
001_DQ060625 TSPGQGDK-- --ITTSKLVA LKDLTGASAD AIIAGTSS-- -----AD--- -GVVTNTGAA SGSTETNSAG
001_DQ060626 TSPGQGDK-- --ITTSKLVA LKDLTGASAD AIIAGTSS-- -----AD--- -GVVTNTGAA SGSTETNSAG
001_DQ060627 TSPGQGDK-- --ITTSKLVA LKDLTGASAD AIIAGTSS-- -----AD--- -GVVTNTGAA SGSTETNSAG
001_AJ300676 TSPGQGDK-- --ITTSKLVA LKDLTGASAD AIIAGTSS-- -----AD--- -GVVTNTGAA SGSTETNSAG
027_R20291 SSPAEGDK-- --VTTSKLVA LKNLTGGTS- AIKVATSS-- -----II--- -GEVENAGTP GAKNTAPSSA
027_CD196 SSPAEGDK-- --VTTSKLVA LKNLTGGTS- AIKVATSS-- -----II--- -GEVENAGTP GAKNTAPSSA
031_DQ060641 TYTTKSI--- --ITKAELKN YYNQLESSKD RILIGNEP-- -----QDTGT KGLI-KADTD GTTAVAAAAP
094_DQ060642 TYTTKSI--- --ITKAELKN YYNQLESSKD RILIGNEP-- -----QDTGT KGLI-KADTD GTTAVAADAP
078_DQ060643 TYVGDADR-- --LTAGDLNT FAKGIADSTE KKIPAAKG-- ---------- ------SNYG VAKTNSGTGK
012_DQ060634 DYNLENKII- --TNQADAEA IVTKLNSLNE KTLIDIAT-- -----KD--T FGMVSKTQDS EGKNVAATKA
012_DQ060635 DYNLENKII- --TNQADAEA IVTKLNSLNE KTLIDIAT-- -----KD--T FGMVSKTQDS EGKNVAATKA
012_AJ291709 DYNLENKII- --TNQADAEA IVTKLNSLNE KTLIDIAT-- -----KD--T FGMVSKTQDS EGKNVAATKA
046_DQ060636 DYNLEKKII- --TNQADAEA IVTKLNSLNE KTLIDIAT-- -----KD--T FGMVSKTQDS EGKNVAATKA
092_DQ060637 DYNLEKKII- --TNQADAEA IVTKLNSLNE KTLIDIAT-- -----KD--T FGMVSKTQDS EGKNVAATKA
017_DQ060640 TYNLATQI-- --ITKAEAEA VLTKLQQYND KVLINSAT-- -----DT--V KGMVSDTQVD SKNVAAN--P
017_AJ300677 TYNLATQI-- --ITKAEAEA VLTKLQQYND KVLINSAT-- -----DT--V KGMVSDTQVD SKNVAAN--P
016_AF478570 AQNLTTGAVI --LNKDTKVT FY-------- ---------- ---------- -----DANEK DSSTPTGDKK
005_DQ060630 AQNLTTGAVI --LNKDTKVT FY-------- ---------- ---------- -----DANEK DSSTPTGDKK
005_DQ060631 AQNLTTGAVI --LNKDTKVT FY-------- ---------- ---------- -----DANEK DSSTPTGDKK
054_DQ060632 AQNLTTGAVI --LNKDTKVT FY-------- ---------- ---------- -----DANEK DSSTPTGDKK
LMW Domain 2
211
010_DQ060633 DTFASYFTVG TVK-TVNGKV ALEINIAEP- ASTVLVKTDA ELTTSPTTQQ KMSFA----- ----------
010_AF478571 DTFASYFTVG TVK-TVNGKV ALEINIAEP- ASTVLVKTDA ELTTSPTTQQ KMSFA----- ----------
002_DQ060628 LKLQKYDTTT SAYVDGATKD EVLAATKAA- VKTSKFTDKT GNNATGTVAI TATYADAVAG AIKLKYDTQA
002_DQ060629 LKLQKYDTTT SAYVDGATKD EVLAATKAA- VKTSKFTDKT GNNATGTVAI TATYADAVAG AIKLKYDTQA
014_DQ060638 LKVKDAVKAT VTT-NGSNKK VLTISAAAG- LSGFSYGTLK DTGASLSDVD AITLD----- ----------
066_DQ060639 LKVKDAVKAT VTT-NGSNKK VLTISAAAG- LSGFSYGTLK DTGASSSDVD AITLD----- ----------
001_DQ060625 TKLAMSAIFD TAY-TDSSET AVKITIKAD- MNDTKFGKAG ETTYSTG--L TFEDG----- ----------
001_DQ060626 TKLAMSAIFD TAY-TDSSET AVKITIKAD- MNDTKFGKAG ETTYSTG--L TFEDG----- ----------
001_DQ060627 TKLAMSAIFD TAY-TDSSET AVKITIKAD- MNDTKFGKAG ETTYSTG--L TFEDG----- ----------
001_AJ300676 TKLAMSAIFD TAY-TDSSET AVKITIKAD- MNDTKFGKAG ETTYSTG--L TFEDG----- ----------
027_R20291 AVMSMSDVFD TAF-TDSTET AVKLTIKDA- MKTKKFGLVD GTTYSTG--L QFADG----- ----------
027_CD196 AVMSMSDVFD TAF-TDSTET AVKLTIKDA- MKTKKFGLVD GTTYSTG--L QFADG----- ----------
031_DQ060641 LKLSDIFTFS YDEVTGVLKA EPTSKVSAGK VQGLKYGNTG ATNYTSG--A EISVP----- ----------
094_DQ060642 LKLSDIFTFS YDEVTGVLKA EPTSKVSAGK VQGLKYGNTG ATNYTSG--A EISVP----- ----------
078_DQ060643 LTTDTEAVIS TSIEGKVEGN NLTISLKDA- --PSKVGVIG ANNDTLA-DV TFAD------ ----------
012_DQ060634 LKVKDVATFG LKS-GGSEDT GYVVEMKAG- AVEDKYGKVG DS--TAG--I AINLP----- ----------
012_DQ060635 LKVKDVATFG LKS-GGSEDT GYVVEMKAG- AVEDKYGKVG DS--TAG--I AINLP----- ----------
012_AJ291709 LKVKDVATFG LKS-GGSEDT GYVVEMKAG- AVEDKYGKVG DS--TAG--I AINLP----- ----------
046_DQ060636 LKVKDVATFG LKS-GGSEDT GYVVEMKAG- AVEDKYGKVG DS--TAG--I AINLP----- ----------
092_DQ060637 LKVKDVATFG LKS-GGSEDT GYVVEMKAG- AVEDKYGKVG DS--TAG--I AINLP----- ----------
017_DQ060640 LKVSDMYTIP SAI-TGSDDS GYSIAKPTEK TTSLLYGTVG DA--TAG--K AITVD----- ----------
017_AJ300677 LKVSDMYTIP SAI-TGSDDS GYSIAKPTEK TTSLLYGTVG DA--TAG--K AITVD----- ----------
016_AF478570 VYSEQILT-- ----TANGNE DYVKTTLKN- LDAGEYAII- DLTYNNA--- ---------- ----------
005_DQ060630 VYSEQTLT-- ----TANGNE DYVKTTLKN- LDAGEYAII- DLTYNNA--- ---------- ----------
005_DQ060631 VYSEQTLT-- ----TANGNE DYVKTTLKN- LDAGEYAII- DLTYNNA--- ---------- ----------
054_DQ060632 VYSEQILT-- ----TANGNE DYVKTTLKN- LDAGEYAII- DLTYNNA--- ---------- ----------

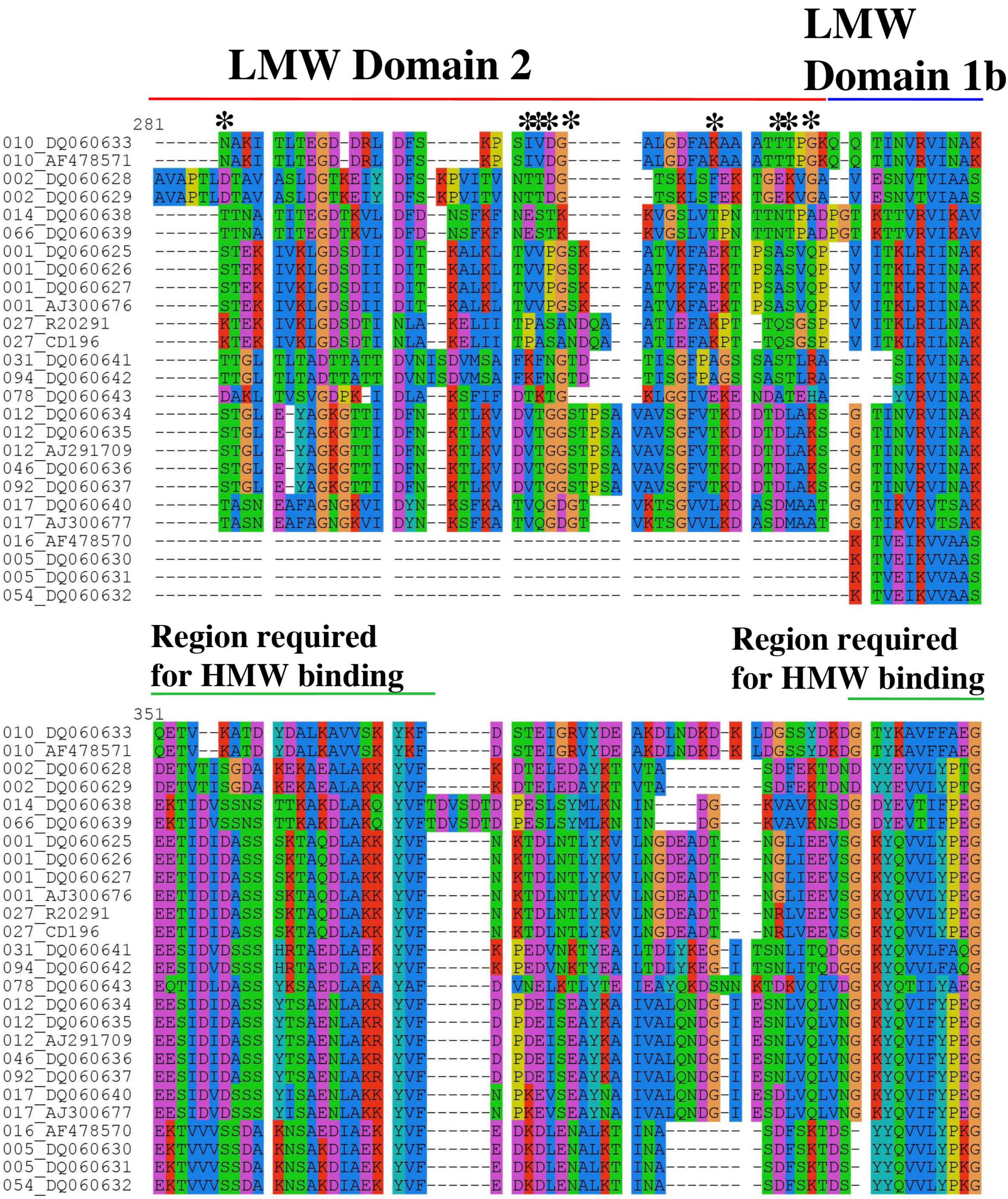
LMW Domain 2
LMW Domain 1b
281
351
Region required for HMW binding
Region required for HMW binding
010_DQ060633
010_AF478571
002_DQ060628
002_DQ060629
014_DQ060638
066_DQ060639
001_DQ060625
001_DQ060626
001_DQ060627
001_AJ300676
027_R20291
027_CD196
031_DQ060641
094_DQ060642
078_DQ060643
012_DQ060634
012_DQ060635
012_AJ291709
046_DQ060636
092_DQ060637
017_DQ060640
017_AJ300677
016_AF478570
005_DQ060630
005_DQ060631
054_DQ060632

Region required for HMW binding

HMW

Region essential for LMW binding

```
              421
010_DQ060633  KRLQGFSSYG KFSTDEAESG LADG------ -NAALKLVIE STDEDDFIDG LKDLKELNNS YSDVESVAGD
010_AF478571  KRLQGFSSYG KFSTDEAESG LADG------ -NAALKLVIE STDEDDFIDG LKDLKELNNS YSDVESVAGD
002_DQ060628  KRLNTASTYA ------S-SN YKPELPTSDR VDTPAIITLR STNKNNLKSA LDELRTYNNS YSNNSVLAGD
002_DQ060629  KRLNTASTYA ------S-SN YKPELPTSDR VDTPAIITLR STNKNNLKSA LDELRTYNNS YSNNSVLAGD
014_DQ060638  KRLNTLSAS- -----SAKTI LGDK------ -DTPAKIVLK ASTTKKLADY IDDLITYNNS YSNVQTVAGS
066_DQ060639  KRLNTLSAS- -----SAKTI LGDK------ -DTPAKIVLK ASTTKKLADY IDDLITYNNS YSNVQTVAGS
001_DQ060625  KRVTTKSAA- -----KA--S IADE------ -NSPVKLTLK SDKKKDLKDY VDDLRTYNNG YSNAIEVAGE
001_DQ060626  KRVTTKSAA- -----KA--S IADE------ -NSPVKLTLK SDKKKDLKDY VDDLRTYNNG YSNAIEVAGE
001_DQ060627  KRVTTKSAA- -----KA--S IADE------ -NSPVKLTLK SDKKKDLKDY VDDLRTYNNG YSNAIEVAGE
001_AJ300676  KRVTTKSAA- -----KA--S IADE------ -NSPVKLTLK SDKKKDLKDY VDDLRTYNNG YSNAIEVAGE
027_R20291    KRVTTKSAA- -----KA--S IADE------ -NSPVKLTLK SDKKKDLKDY VDDLRTYNNG YSNAIEVAGE
027_CD196     KRVTTKSAA- -----KA--S IADE------ -NSPVKLTLK SDKKKDLKDY VDDLRTYNNG YSNAIEVAGE
031_DQ060641  KRLTTKGAT- -----GT--- LADE------ -NSPLKVTIK ADKVKDLKDY VEDLKNANNG YSNSVVVAGE
094_DQ060642  KRLTTKGAT- -----GT--- LADE------ -NSPLKVTIK ADKVKDLKDY VEDLKNANNG YSNSVVVAGE
078_DQ060643  KRLTTKSAT- -----QA-SK LADE------ -NSPLKLVIK ADKLKDLKDY VEDLRNANNG YSNTVTVAGD
012_DQ060634  KRLETKSAN- -----DT--- IASQ------ -DTPAKVVIK ANKLKDLKDY VDDLKTYNNT YSNVVTVAGE
012_DQ060635  KRLETKSAN- -----DT--- IASQ------ -DTPAKVVIK ANKLKDLKDY VDDLKTYNNT YSNVVTVAGE
012_AJ291709  KRLETKSAN- -----DT--- IASQ------ -DTPAKVVIK ANKLKDLKDY VDDLKTYNNT YSNVVTVAGE
046_DQ060636  KRLETKSAN- -----DT--- IASQ------ -DTPAKVVIK ANKLKDLKDY VDDLKTYNNT YSNVVTVAGE
092_DQ060637  KRLETKSAN- -----DT--- IASQ------ -DTPAKVVIK ANKLKDLKDY VDDLKTYNNT YSNVVTVAGE
017_DQ060640  KRLETKSAD- ---------I IADA------ -DSPAKITIK ANKLKDLKDY VDDLKTYNNT YSNVVTVAGE
017_AJ300677  KRLETKSAD- ---------I IADA------ -DSPAKITIK ANKLKDLKDY VDDLKTYNNT YSNVVTVAGE
016_AF478570  KRLQGFSTYR ------A-TN YNEGTAYG-- -NTPVILTLK STSKSNLKTA VEELQKLNAS YSNTTTLAGD
005_DQ060630  KRLQGFSTYR ------A-TN YNEGTAYG-- -NTPVILTLK STSKSNLKTA VEELQKLNAS YSNTTTLAGD
005_DQ060631  KRLQGFSTYR ------A-TN YNEGTAYG-- -NTPVILTLK STSKSNLKTA VEELQKLNAS YSNTTTLAGD
054_DQ060632  KRLQGFSTYR ------A-TN YNEGTAYG-- -NTPVILTLK STSKSNLKTA VEELQKLNAS YSNTTTLAGD
```

HMW

```
              491                   *
010_DQ060633  DRIETAIELS KSYY-NNS-R SNNSDTLYTG AV--DNVVLV GSQAIVDGLV AGPLAAEKEG PLLLSSKDKL
010_AF478571  DRIETAIELS KSYY-NNS-R SNNSDTLYTG AV--DNVVLV GSQAIVDGLV AGPLAAEKEG PLLLSSKDKL
002_DQ060628  DRIETAIEIS KDSY-NAD-- ----GGIKGD YVEANEVVLV GSQSIVDGLV ASPLAAVKDA PLLLTSKDKL
002_DQ060629  DRIETAIEIS KDSY-NAD-- ----GGIKGD YVEANEVVLV GSQSIVDGLV ASPLAAVKDA PLLLTSKDKL
014_DQ060638  DRIETAIELS RKYY-NST-- --DKNALYGD PV--NNVVLV GSQAIVDGLV ASPLAAEKDA PLLLSSKDKL
066_DQ060639  DRIETAIELS RKYY-NST-- --DKNALYGD PV--NNVVLV GSQAIVDGLV ASPLAAEKDA PLLLSSKDKL
001_DQ060625  DRIETAIALS QKYY-NSD-- --DENAIFRD SV--DNVVLV GGNAIVDGLV ASPLASEKKA PLLLTSKDKL
001_DQ060626  DRIETAIALS QKYY-NSD-- --DENAIFRD SV--DNVVLV GGNAIVDGLV ASPLASEKKA PLLLTSKDKL
001_DQ060627  DRIETAIALS QKYY-NSD-- --DENAIFRD SV--DNVVLV GGNAIVDGLV ASPLASEKKA PLLLTSKDKL
001_AJ300676  DRIETAIALS QKYY-NSD-- --DENAIFRD SV--DNVVLV GGNAIVDGLV ASPLASEKKA PLLLTSKDKL
027_R20291    DRIETAIALS QKYY-NSD-- --DENAIFRD SV--DNVVLV GGNAIVDGLV ASPLASEKKA PLLLTSKDKL
027_CD196     DRIETAIALS QKYY-NSD-- --DENAIFRD SV--DNVVLV GGNAIVDGLV ASPLASEKKA PLLLTSKDKL
031_DQ060641  DRIETAIELS SKYY-NSD-- --DDNAITKD PV--NNVVLV GSQAVVDGLV ASPLASEKRA PLLLTSAGKL
094_DQ060642  DRIETAIELS SKYY-NSD-- --DDNAITKD PV--NNVVLV GSQAVVDGLV ASPLASEKRA PLLLTSAGKL
078_DQ060643  DRIETAIELS SKYYNNSD-- --EDNAITED AV--NNVVLV GSQAIVDGLV ASPLASEKKA PLLLTSKDKL
012_DQ060634  DRIETAIELS SKYY-NSD-- --DKNAITDK AV--NDIVLV GSTSIVDGLV ASPLASEKTA PLLLTSKDKL
012_DQ060635  DRIETAIELS SKYY-NSD-- --DKNAITDK AV--NDIVLV GSTSIVDGLV ASPLASEKTA PLLLTSKDKL
012_AJ291709  DRIETAIELS SKYY-NSD-- --DKNAITDK AV--NDIVLV GSTSIVDGLV ASPLASEKTA PLLLTSKDKL
046_DQ060636  DRIETAIELS SKYY-NSD-- --DKNAITDK AV--NDIVLV GSTSIVDGLV ASPLASEKTA PLLLTSKDKL
092_DQ060637  DRIETAIELS SKYY-NSD-- --DKNAITDK AV--NDIVLV GSTSIVDGLV ASPLASEKTA PLLLTSKDKL
017_DQ060640  DRIETAIELS SKYY-NSD-- --DKNAITDD AV--NNIVLV GSTSIVDGLV ASPLASEKTA PLLLTSKDKL
017_AJ300677  DRIETAIELS SKYY-NSD-- --DKNAITDD AV--NNIVLV GSTSIVDGLV ASPLASEKTA PLLLTSKDKL
016_AF478570  DRIQTAIEIS KEYY-NNDGE KQDHSADVKE NV--KNVVLV GANALVDGLV AAPLAAEKDA PLLLTSKDKL
005_DQ060630  DRIQTAIEIS KEYY-NNDGE KSDHSADVKE NV--KNVVLV GANALVDGLV AAPLAAEKDA PLLLTSKDKL
005_DQ060631  DRIQTAIEIS KEYY-NNDGE KSDHSADVKE NV--KNVVLV GANALVDGLV AAPLAAEKDA PLLLTSKDKL
054_DQ060632  DRIQTAIEIS KEYY-NNDGE KQDHSADVKE NV--KNVVLV GANALVDGLV AAPLAAEKDA PLLLTSKDKL
```

HMW

```
            561
010_DQ060633 DNNVKNEIKR VMGLSSTNSI DSKKKVYIVG GENSVSKDVQ KAIEDMGVKV ERLSGDDRYA TSLKIADKVE
010_AF478571 DNNVKNEIKR VMGLSSTNSI DSKKKVYLAG GVNSVSKDVQ KAIEDMGVKV ERLSGDDRYA TSLKIADKVE
002_DQ060628 DSSVKSEIKR VMGLDDKTGI TSKKTVYIAG GENSVSKEVA NELKDMGLKV ERLSGDDRYA TSLEIADEIG
002_DQ060629 DSSVKSEIKR VMGLDDKTGI TSKKTVYIAG GENSVSKEVA NELKDMGLKV ERLSGDDRYA TSLEIADEIG
014_DQ060638 DSSTRAEIKR VMDLNSSTGI KNNKEVFIAG GVNSISKDVE NELKDMGLKV TRLSGDDRYE TSLAIADEID
066_DQ060639 DSSTRAEIKR VMDLNSSTGI KNNKEVFIAG GVNSISKDVE NELKDMGLKV TRLSGDDRYE TSLAIADEID
001_DQ060625 DSSVKAEIKR VMNIKSTTGI NTSKKVYLAG GVNSISKEVE NELKDMGLKV TRLAGDDRYE TSLKIADEVG
001_DQ060626 DSSVKAEIKR VMNIKSTTGI NTSKKVYLAG GVNSISKEVE NELKDMGLKV TRLAGDDRYE TSLKIADEVG
001_DQ060627 DSSVKAEIKR VMNIKSTTGI NTSKKVYLAG GVNSISKEVE NELKDMGLKV TRLAGDDRYE TSLKIADEVG
001_AJ300676 DSSVKAEIKR VMNIKSTTGI NTSKKVYLAG GVNSISKEVE NELKDMGLKV TRLAGDDRYE TSLKIADEVG
027_R20291   DSSVKAEIKR VMNIKSTTGI NTSKKVYLAG GVNSISKEVE NELKDMGLKV TRLAGDDRYE TSLKIADEVG
027_CD196    DSSVKAEIKR VMNIKSTTGI NTSKKVYLAG GVNSISKEVE NELKDMGLKV TRLAGDDRYE TSLKIADEVG
031_DQ060641 DSSVKAELKR VMDLKSTTGV NTSKKVYLAG GVNSISKDVE NELKDMGLKV TRLSGDDRYE TSLAIADEIG
094_DQ060642 DSSVKAELKR VMDLKSTTGV NTSKKVYLAG GVNSISKDVE NELKDMGLKV TRLSGDDRYE TSLAIADEIG
078_DQ060643 DSNVKSEIKR VMNLKTTTGI NNSKKVYLAG GVNSISKEVE NELKDMGLKV TRLSGDDRYA TSLEIADEIG
012_DQ060634 DSSVKSEIKR VMNLKSDTGI NTSKKVYLAG GVNSISKDVE NELKNMGLKV TRLSGEDRYE TSLAIADEIG
012_DQ060635 DSSVKSEIKR VMNLKSDTGI NTSKKVYLAG GVNSISKDVE NELKNMGLKV TRLSGEDRYE TSLAIADEIG
012_AJ291709 DSSVKSEIKR VMNLKSDTGI NTSKKVYLAG GVNSISKDVE NELKNMGLKV TRLSGEDRYE TSLAIADEIG
046_DQ060636 DSSVKSEIKR VMNLKSDTGI NTSKKVYLAG GVNSISKDVE NELKNMGLKV TRLSGEDRYE TSLAIADEIG
092_DQ060637 DSSVKSEIKR VMNLKSDTGI NTSKKVYLAG GVNSISKDVE NELKNMGLKV TRLSGEDRYE TSLAIADEIG
017_DQ060640 DSSVKSEIKR VMNLKSDTGI NTSKKVYLAG GVNSISKDVE NELKNMGLKV TRLSGEDRYE TSLAIADEIG
017_AJ300677 DSSVKSEIKR VMNLKSDTGI NTSKKVYLAG GVNSISKDVE NELKNMGLKV TRLSGEDRYE TSLAIADEIG
016_AF478570 DSSVKSEIKR VLDLKTSTEV -TGKTVYLAG GVNSVSKEVV TELESMGLKV ERFSGDDRYE TSLKIADEIG
005_DQ060630 DSSVKSEIKR VLDLKTSTEV -TGKTVYIAG GVNSVSKEVV TELESMGLKV ERFSGDDRYE TSLKIADEIG
005_DQ060631 DSSVKSEIKR VLDLKTSTEV -TGKTVYIAG GVNSVSKEVV TELESMGLKV ERFSGDDRYE TSLKIADEIG
054_DQ060632 DSSVKSEIKR VLDLKTSTEV -TGKTVYIAG GVNSVSKEVV TELESMGLKV ERFSGDDRYE TSLKIADEIG
```

HMW

```
            631
010_DQ060633 LNDKDKAFVV GGTGLADAMS IAPVASQLVG ---------K EATPIVVVDG KADKLSSDAS DFLDSAKEVD
010_AF478571 LNDKDKAFVV GGTGLADAMS IAPVASQLVG ---------K EATPIVVVDG KADKLSSDAS DFLDSAKEVD
002_DQ060628 L-NHNKVFVV GGTGLADAMS IASVAS---- ---------N KEMPIVVVDG KGKDLSTDAK DFIGSA-YVD
002_DQ060629 L-NHNKVFVV GGTGLADAMS IASVAS---- ---------N KEMPIVVVDG KGKDLSTDAK DFIGSA-YVD
014_DQ060638 I--NDKAYVV GGTGLADAMS IAPVASQIKD ---------G EATPIVVVDG KSDKLSKEAE DFLDDA-QVD
066_DQ060639 I--NDKAYVV GGTGLADAMS IAPVASQIKD ---------G EATPIVVVDG KSDKLSKEAE DFLDDA-QVD
001_DQ060625 L-DNDKAFVV GGTGLADAMS IAPVASQLRN ANGKMDLADG DATPIVVVDG KAKTINDDVK DFLDDS-QVD
001_DQ060626 L-DNDKAFVV GGTGLADAMS IAPVASQLRN ANGKMDLADG DATPIVVVDG KAKTINDDVK DFLDDS-QVD
001_DQ060627 L-DNDKAFVV GGTGLADAMS IAPVASQLRN ANGKMDLADG DATPIVVVDG KAKTINDDVK DFLDDS-QVD
001_AJ300676 L-DNDKAFVV GGTGLADAMS IAPVASQLRN ANGKMDLADG DATPIVVVDG KAKTINDDVK DFLDDS-QVD
027_R20291   L-DNDKAFVV GGTGLADAMS IAPVASQLRN ANGKMDLADG DATPIVVVDG KAKTINDDVK DFLDDS-QVD
027_CD196    L-DNDKAFVV GGTGLADAMS IAPVASQLRN ANGKMDLADG DATPIVVVDG KAKTINDDVK DFLDDS-QVD
031_DQ060641 L-DNDKAFVV GGTGLADAMS IAPVASQLRN SNGELDL-KG DATPIVVVDG KAKDINSEVK DFLDDS-QVD
094_DQ060642 L-DNDKAFVV GGTGLADAMS IAPVASQLRN SNGELDL-KG DATPIVVVDG KAKDINSEVK DFLDDS-QVD
078_DQ060643 L-DDDKAFVV GGTGLADAMS IAPVASQLNE --------KG DATPIVVVDG KAKELSSAAE DFLDDS-QVD
012_DQ060634 L-DNDKAFVV GGTGLADAMS IAPVASQLKD ---------G DATPIVVVDG KAKEISDDAK SFLGTS-DVD
012_DQ060635 L-DNDKAFVV GGTGLADAMS IAPVASQLKD ---------G DATPIVVVDG KAKEISDDAK SFLGTS-DVD
012_AJ291709 L-DNDKAFVV GGTGLADAMS IAPVASQLKD ---------G DATPIVVVDG KAKEISDDAK SFLGTS-DVD
046_DQ060636 L-DNDKAFVV GGTGLADAMS IAPVASQLKD ---------G DATPIVVVDG KAKEISDDAK SFLGTS-DVD
092_DQ060637 L-DNDKAFVV GGTGLADAMS IAPVASQLKD ---------G DATPIVVVDG KAKEISDDAK SFLGTS-DVD
017_DQ060640 L-DNDKAFVV GGTGLADAMS IAPVASQLKD ---------G DATPIVVVDG KAKEISDDAK SFLGTS-DVD
017_AJ300677 L-DNDKAFVV GGTGLADAMS IAPVASQLKD ---------G DATPIVVVDG KAKEISDDAK SFLGTS-DVD
016_AF478570 L-DNDKAYVV GGTGLADAMS IASVASTKLD GNGVVDKTNG HATPIVVVDG KADKISDDLD SFLGSA-DVD
005_DQ060630 L-DNDKAYVV GGTGLADAMS IASVASTKLD GNGVVDRTNG HATPIVVVDG KADKISDDLD SFLGSA-DVD
005_DQ060631 L-DNDKAYVV GGTGLADAMS IASVASTKLD GNGVVDRTNG HATPIVVVDG KADKISDDLD SFLGSA-DVD
054_DQ060632 L-DNDKAYVV GGTGLADAMS IASVASTKLD GNGVVDRTNG HATPIVVVDG KADKISDDLD SFLGSA-DVD
```

HMW

```
             701
010_DQ060633 IIGGENSVSN KVKDSIKDAI GRSVDRISGD DRQATNAEVI KE--YY---- ---------- ----ENDPKN
010_AF478571 IIGGENSVSN KVKDSIKDAI GRSVDRISGD DRQATNAEVI KE--YY---- ---------- ----ENDPKN
002_DQ060628 IIGGKSSVSE DMEDAIDDAT GKSPERVSGD DRQDTNAEVI KT--YF---- --------EK DNSDSVISTG
002_DQ060629 IIGGKSSVSE DMEDAIDDAT GKSPERVSGD DRQDTNAEVI KT--YF---- --------EK DNSDSVISTG
014_DQ060638 IIGGENSVSA KMEDYIDDAT GKSPERISGA DRQATNAEVI KE--YF---- ---------- ------DKDG
066_DQ060639 IIGGENSVSA KMEDYIDDAT GKSPERISGA DRQATNAEVI KE--YF---- ---------- ------DKDG
001_DQ060625 IIGGENSVSK DVENAIDDAT GKSPDRYSGD DRQATNAKVI KESSYY---- ---------- -QDNLNNDKK
001_DQ060626 IIGGENSVSK DVENAIDDAT GKSPDRYSGD DRQATNAKVI KESSYY---- ---------- -QDNLNNDKK
001_DQ060627 IIGGENSVSK DVENAIDDAT GKSPDRYSGD DRQATNAKVI KESSYY---- ---------- -QDNLNNDKK
001_AJ300676 IIGGENSVSK DVENAIDDAT GKSPDRYSGD DRQATNAKVI KESSYY---- ---------- -QDNLNNDKK
027_R20291   IIGGENSVSK DVENAIDDAT GKSPDRYSGD DRQATNAKVI KESSYY---- ---------- -QDNLNNDKK
027_CD196    IIGGENSVSK DVENAIDDAT GKSPDRYSGD DRQATNAKVI KESSYY---- ---------- -QDNLNNDKK
031_DQ060641 IIGGVNSVSK EVMEAIDDAT GKSPERYSGE DRQATNAKVI KEDDFF---- ---------- ------KNGE
094_DQ060642 IIGGVNSVSK EVMEAIDDAT GKSPERYSGE DRQATNAKVI KEDDFF---- ---------- ------KNGE
078_DQ060643 IIGGKNSVSK DMEDAIDDAT GKSPNRVSGD DRQETNAEVL KESDYF---- ---------- ------PDG-
012_DQ060634 IIGGKNSVSK EIEESIDSAT GKTPDRISGD DRQATNAEVL KEDDYF---- ---------- ------TDGE
012_DQ060635 IIGGKNSVSK EIEESIDSAT GKTPDRISGD DRQATNAEVL KEDDYF---- ---------- ------TDGE
012_AJ291709 IIGGKNSVSK EIEESIDSAT GKTPDRISGD DRQATNAEVL KEDDYF---- ---------- ------TDGE
046_DQ060636 IIGGKNSVSK EIEESIDSAT GKTPDRISGD DRQATNAEVL KEDDYF---- ---------- ------TDGE
092_DQ060637 IIGGKNSVSK EIEESIDSAT GKTPDRISGD DRQATNAEVL KEDDYF---- ---------- ------TDGE
017_DQ060640 IIGGKNSVSK EIEESIDSAT GKTPDRISGD DRQATNAEVL KEDDYF---- ---------- ------KDGE
017_AJ300677 IIGGKNSVSK EIEESIDSAT GKTPDRISGD DRQATNAEVL KEDDYF---- ---------- ------KDGE
016_AF478570 IIGGFASVSE KMEEAISDAT GKGVTRVKGD DRQDTNSEVI KT--YYANDT EIAKAAVLDK DSGASSSDAG
005_DQ060630 IIGGFASVSE KMEEAISDAT GKGVTRVKGD DRQDTNSEVI KT--YYANDT EIAKAAVLDK DSGASSSDAG
005_DQ060631 IIGGFASVSE KMEEAISDAT GKGVTRVKGD DRQDTNSEVI KT--YYANDT EIAKAAVLDK DSGASSSDAG
054_DQ060632 IIGGFASVSE KMEEAISDAT GKGVTRVKGD DRQDTNSEVI KT--YYANDT EIAKAAVLDK DSGASSSDAG
```

HMW

```
             771
010_DQ060633 VKNIFVAKDG STKEDQLVDA LAAGAIAGNL GLSAGEDE-- ---------- --------VS PAPIVLATDN
010_AF478571 VKNIFVAKDG STKEDQLVDA LAAGAIAGNL GLSAGEDE-- ---------- --------VS PAPIVLATDN
002_DQ060628 VKNFYVAKDG STKEDQLVDA LAIAAVAGH- ---------- ---------- ---------N EAPIVLATDS
002_DQ060629 VKNFYVAKDG STKEDQLVDA LAIAAVAGH- ---------- ---------- ---------N EAPIVLATDS
014_DQ060638 VSNYFLAKDG STKEDQLVDA LAAAAVAGNY GSKHNEDGDI TTD------- --------AS PAPIILATDN
066_DQ060639 VSNYFLAKDG STKEDQLVDA LAAAAVAGNY GSKHNEDGDI TTD------- --------AS PAPIILATDN
001_DQ060625 VVNFFVAKDG STKEDQLVDA LAAAPVAANF GVTLNSDGKP VDKDGKVLTG SDNDKNKLVS PAPIVLATDS
001_DQ060626 VVNFFVAKDG STKEDQLVDA LAAAPVAANF GVTLNSDGKP VDKDGKVLTG SDNDKNKLVS PAPIVLATDS
001_DQ060627 VVNFFVAKDG STKEDQLVDA LAAAPVAANF GVTLNSDGKP VDKDGKVLTG SDNDKNKLVS PAPIVLATDS
001_AJ300676 VVNFFVAKDG STKEDQLVDA LAAAPVAANF GVTLNSDGKP VDKDGKVLTG SDNDKNKLVS PAPIVLATDS
027_R20291   VVNFFVAKDG STKEDQLVDA LAAAPVAANF GVTLNSDGKP VDKDGKVLTG SDNDKNKLVS PAPIVLATDS
027_CD196    VVNFFVAKDG STKEDQLVDA LAAAPVAANF GVTLNSDGKP VDKDGKVLTG SDNDKNKLVS PAPIVLATDS
031_DQ060641 VTNFFVAKDG STKEDQLVDA LAGAAIAGNF GVTVDNEGKP TVADKK---- --------AS PAPIVLATDS
094_DQ060642 VTNFFVAKDG STKEDQLVDA LAGAAIAGNF GVTVDNEGKP TVADKK---- --------AS PAPIVLATDS
078_DQ060643 AVNYFVAKDG STKEDQLVDA LAAAPVAANF GRTYNIK--- ---------- -DNDSSGTVS PAPIILATDS
012_DQ060634 VVNYFVAKDG STKEDQLVDA LAAAPIAGRF KE-------- ---------- ---------S PAPIILATDT
012_DQ060635 VVNYFVAKDG STKEDQLVDA LAAAPIAGRF KE-------- ---------- ---------S PAPIILATDT
012_AJ291709 VVNYFVAKDG STKEDQLVDA LAAAPIAGRF KE-------- ---------- ---------S PAPIILATDT
046_DQ060636 VVNYFVAKDG STKEDQLVDA LAAAPIAGRF KE-------- ---------- ---------S PAPIILATDT
092_DQ060637 VVNYFVAKDG STKEDQLVDA LAAAPIAGRF KE-------- ---------- ---------S PAPIILATDT
017_DQ060640 VVNYFVAKDG STKEDQLVDA LAAAPIAGRF KE-------- ---------- ---------S PAPIILATDT
017_AJ300677 VVNYFVAKDG STKEDQLVDA LAAAPIAGRF KE-------- ---------- ---------S PAPIILATDT
016_AF478570 VFNFYVAKDG STKEDQLVDA LAVGAVAGY- ---------- ---------- ---------K LAPVVLATDS
005_DQ060630 VFNFYVAKDG STKEDQLVDA LAVGAVAGY- ---------- ---------- ---------K LAPVVLATDS
005_DQ060631 VFNFYVAKDG STKEDQLVDA LAVGAVAGY- ---------- ---------- ---------K LAPVVLATDS
054_DQ060632 VFNFYVAKDG STKEDQLVDA LAVGAVAGY- ---------- ---------- ---------K LAPVVLATDS
```

HMW

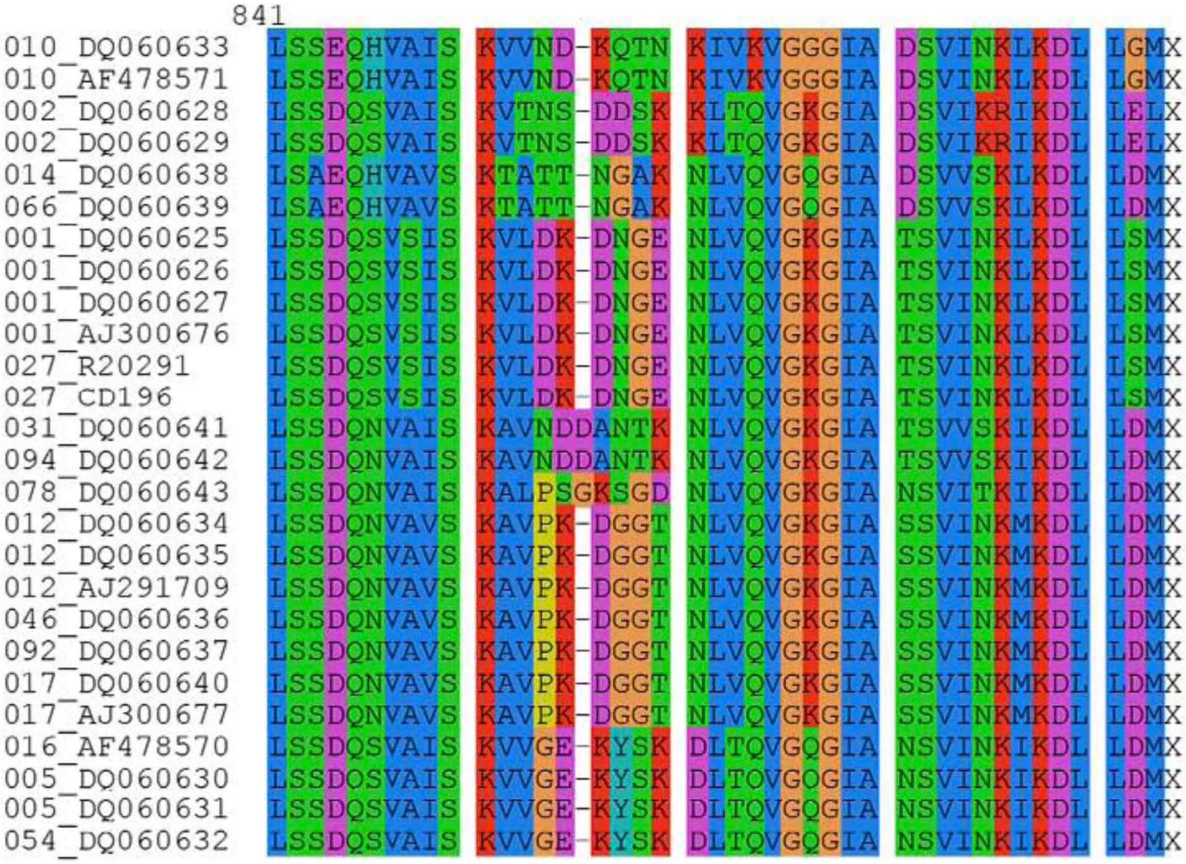
841
010_DQ060633 LSSEQHVAIS KVVND-KQTN KIVKVGGGIA DSVINKLKDL LGMX
010_AF478571 LSSEQHVAIS KVVND-KQTN KIVKVGGGIA DSVINKLKDL LGMX
002_DQ060628 LSSDQSVAIS KVTNS-DDSK KLTQVGKGIA DSVIKRIKDL LELX
002_DQ060629 LSSDQSVAIS KVTNS-DDSK KLTQVGKGIA DSVIKRIKDL LELX
014_DQ060638 LSAEQHVAVS KTATT-NGAK NLVQVGQGIA DSVVSKLKDL LDMX
066_DQ060639 LSAEQHVAVS KTATT-NGAK NLVQVGQGIA DSVVSKLKDL LDMX
001_DQ060625 LSSDQSVSIS KVLDK-DNGE NLVQVGKGIA TSVINKLKDL LSMX
001_DQ060626 LSSDQSVSIS KVLDK-DNGE NLVQVGKGIA TSVINKLKDL LSMX
001_DQ060627 LSSDQSVSIS KVLDK-DNGE NLVQVGKGIA TSVINKLKDL LSMX
001_AJ300676 LSSDQSVSIS KVLDK-DNGE NLVQVGKGIA TSVINKLKDL LSMX
027_R20291 LSSDQSVSIS KVLDK-DNGE NLVQVGKGIA TSVINKLKDL LSMX
027_CD196 LSSDQSVSIS KVLDK-DNGE NLVQVGKGIA TSVINKLKDL LSMX
031_DQ060641 LSSDQNVAIS KAVNDDANTK NLVQVGKGIA TSVVSKIKDL LDMX
094_DQ060642 LSSDQNVAIS KAVNDDANTK NLVQVGKGIA TSVVSKIKDL LDMX
078_DQ060643 LSSDQNVAIS KALPSGKSGD NLVQVGKGIA NSVITKIKDL LDMX
012_DQ060634 LSSDQNVAVS KAVPK-DGGT NLVQVGKGIA SSVINKMKDL LDMX
012_DQ060635 LSSDQNVAVS KAVPK-DGGT NLVQVGKGIA SSVINKMKDL LDMX
012_AJ291709 LSSDQNVAVS KAVPK-DGGT NLVQVGKGIA SSVINKMKDL LDMX
046_DQ060636 LSSDQNVAVS KAVPK-DGGT NLVQVGKGIA SSVINKMKDL LDMX
092_DQ060637 LSSDQNVAVS KAVPK-DGGT NLVQVGKGIA SSVINKMKDL LDMX
017_DQ060640 LSSDQNVAVS KAVPK-DGGT NLVQVGKGIA SSVINKMKDL LDMX
017_AJ300677 LSSDQNVAVS KAVPK-DGGT NLVQVGKGIA SSVINKMKDL LDMX
016_AF478570 LSSDQSVAIS KVVGE-KYSK DLTQVGQGIA NSVINKIKDL LDMX
005_DQ060630 LSSDQSVAIS KVVGE-KYSK DLTQVGQGIA NSVINKIKDL LDMX
005_DQ060631 LSSDQSVAIS KVVGE-KYSK DLTQVGQGIA NSVINKIKDL LDMX
054_DQ060632 LSSDQSVAIS KVVGE-KYSK DLTQVGQGIA NSVINKIKDL LDMX

B

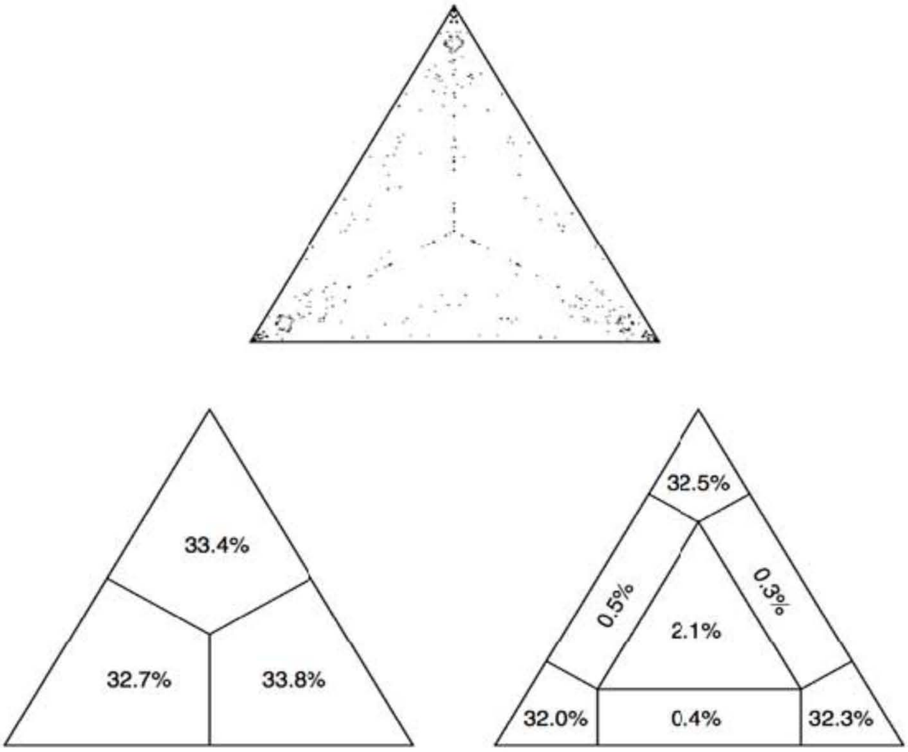
33.4%
32.7%
33.8%
32.5%
0.5%
0.3%
2.1%
32.0%
0.4%
32.3%
